# IFN-γ immune priming of macrophages *in vivo* induces prolonged STAT1 binding and protection against *Cryptococcus neoformans*

**DOI:** 10.1371/journal.ppat.1007358

**Published:** 2018-10-10

**Authors:** Chrissy M. Leopold Wager, Camaron R. Hole, Althea Campuzano, Natalia Castro-Lopez, Hong Cai, Marley C. Caballero Van Dyke, Karen L. Wozniak, Yufeng Wang, Floyd L. Wormley

**Affiliations:** 1 Department of Biology, The University of Texas at San Antonio, San Antonio, TX, United States of America; 2 The South Texas Center for Emerging Infectious Diseases, The University of Texas at San Antonio, San Antonio, TX, United States of America; Louisiana State University Health Sciences Center New Orleans, UNITED STATES

## Abstract

Development of vaccines against opportunistic infections is difficult as patients most at risk of developing disease are deficient in aspects of the adaptive immune system. Here, we utilized an experimental immunization strategy to induce innate memory in macrophages *in vivo*. Unlike current trained immunity models, we present an innate memory-like phenotype in macrophages that is maintained for at least 70 days post-immunization and results in complete protection against secondary challenge in the absence of adaptive immune cells. RNA-seq analysis of *in vivo* IFN-γ primed macrophages revealed a rapid up-regulation of IFN-γ and STAT1 signaling pathways following secondary challenge. The enhanced cytokine recall responses appeared to be pathogen-specific, dependent on changes in histone methylation and acetylation, and correlated with increased STAT1 binding to promoter regions of genes associated with protective anti-fungal immunity. Thus, we demonstrate an alternative mechanism to induce macrophage innate memory *in vivo* that facilitates pathogen-specific vaccine-mediated immune responses.

## Author summary

Fungal infections are a significant global health problem that can affect anyone, however, individuals with a weakened immune system are most at risk. *Cryptococcus neoformans* infections can progress to meningitis in immune compromised individuals accounting for nearly 220,000 new cases annually, resulting in 181,000 deaths. Vaccine strategies tend to target CD4^+^ T cells for the generation of protective memory responses. However, immune compromised individuals have decreased numbers of these adaptive cells, providing a challenge for anti-fungal vaccine design. Here, we define a cellular mechanism by which macrophages, an innate cell population, generate protective immune responses against *C*. *neoformans* following initial exposure to a *C*. *neoformans* strain that secretes IFN-γ. We determined that the macrophages primed *in vivo* have heightened proinflammatory cytokine responses upon secondary exposure to *C*. *neoformans* in a manner that is mTOR-independent, yet dependent on histone modification dynamics. We show that IFN-γ primed macrophages can maintain STAT1 binding to the promoter regions of key proinflammatory genes long after the initial exposure. Remarkably, our studies show long-lived, cryptococcal-specific protective immunity *in vivo*. The results presented herein demonstrate that innate cell populations, namely macrophages, can be utilized as vaccine targets to protect against cryptococcal infections in immune compromised populations.

## Introduction

Vaccines in clinical use today were designed from the onset to generate antigen-specific memory T and/or B cell (antibody) immune responses (reviewed in [1–3]). However, vaccines designed to elicit T cell and/or antibody-mediated immune responses are predicted to be ineffective in patients rendered immune compromised due to diseases such as HIV/AIDS or immunosuppressive therapies to prevent solid organ transplant rejection or ameliorate various autoimmune diseases. Novel approaches in vaccine design are needed to induce protective immunity in the increasing population of immunocompromised individuals.

The ability of innate immune cells to possess memory like-responses upon re-exposure to an antigen has been documented in plants, insects, and more recently, mammals. Memory-like responses by innate immune cells have been termed innate memory or “trained” immunity (reviewed in [4–7]). Trained immunity occurs independent of B and T cell adaptive responses, increases resistance of the host to reinfection, and involves cells including monocytes, macrophages, and NK cells [4]. Studies have shown that following the initial antigen stimulation, innate cells are capable of enhanced pro-inflammatory cytokine recall responses following secondary exposure to intracellular pathogens at 7 days post training [8, 9]. Thus, this concept suggests that immunization strategies that drive memory-like innate immune responses may provide a novel and effective mechanism for inducing vaccine-mediated protection in immunocompromised patients.

Our laboratory has utilized a fungal vaccine model that delivers interferon-γ (IFN-γ) *in vivo* to demonstrate the induction of protective immunity against disease in vaccinated B cell-deficient mice and CD4^+^/CD8^+^ T cell-depleted mice [10, 11]. These studies reveal that protective immunity can be achieved in hosts devoid of immune cells traditionally considered necessary for adaptive immunity and provide proof-of-concept that protection can be achieved in immunocompromised patients. However, the effector cell population and mechanism responsible for protection is unknown.

In the current studies, we sought to determine the mechanism(s) underlying innate memory using our protective fungal vaccine model. We observed that protectively immunized B cell KO mice that were subsequently depleted of T cells, neutrophils, and natural killer (NK) cells were protected against challenge with the opportunistic fungal pathogen *Cryptococcus neoformans*; a significant cause of morbidity and mortality in HIV^+^/AIDS patients world-wide [12–14]. Macrophages from protectively immunized mice displayed enhanced antigen-specific cytokine recall responses using a mechanism distinct from the established trained immunity paradigm. The transcriptome of pulmonary macrophages isolated from the protectively immunized mice early post-challenge demonstrated increased expression of genes associated with protective anti-fungal immune responses; namely, genes associated with the signal transducer and activator of transcription 1 (STAT1) signaling pathway. Enhanced binding of STAT1 to the promotor regions of known IFN-γ-induced genes was observed in immunized, unchallenged pulmonary macrophages, correlating with these macrophages’ ability to quickly respond to secondary challenge. Thus, IFN-γ priming of macrophages *in vivo* resulted in the establishment of antigen-specific innate memory-like responses through 70 days post-immunization and provided complete protection against secondary challenge in the absence of adaptive immune cells. Altogether, our studies demonstrate the feasibility of vaccine strategies designed to enhance innate immune responses against specific pathogens to provide protection against diseases that target immunocompromised individuals.

## Results

### Adaptive immunity is not required for protective responses following immunization with *C*. *neoformans* strain H99γ

**Fig 1:**
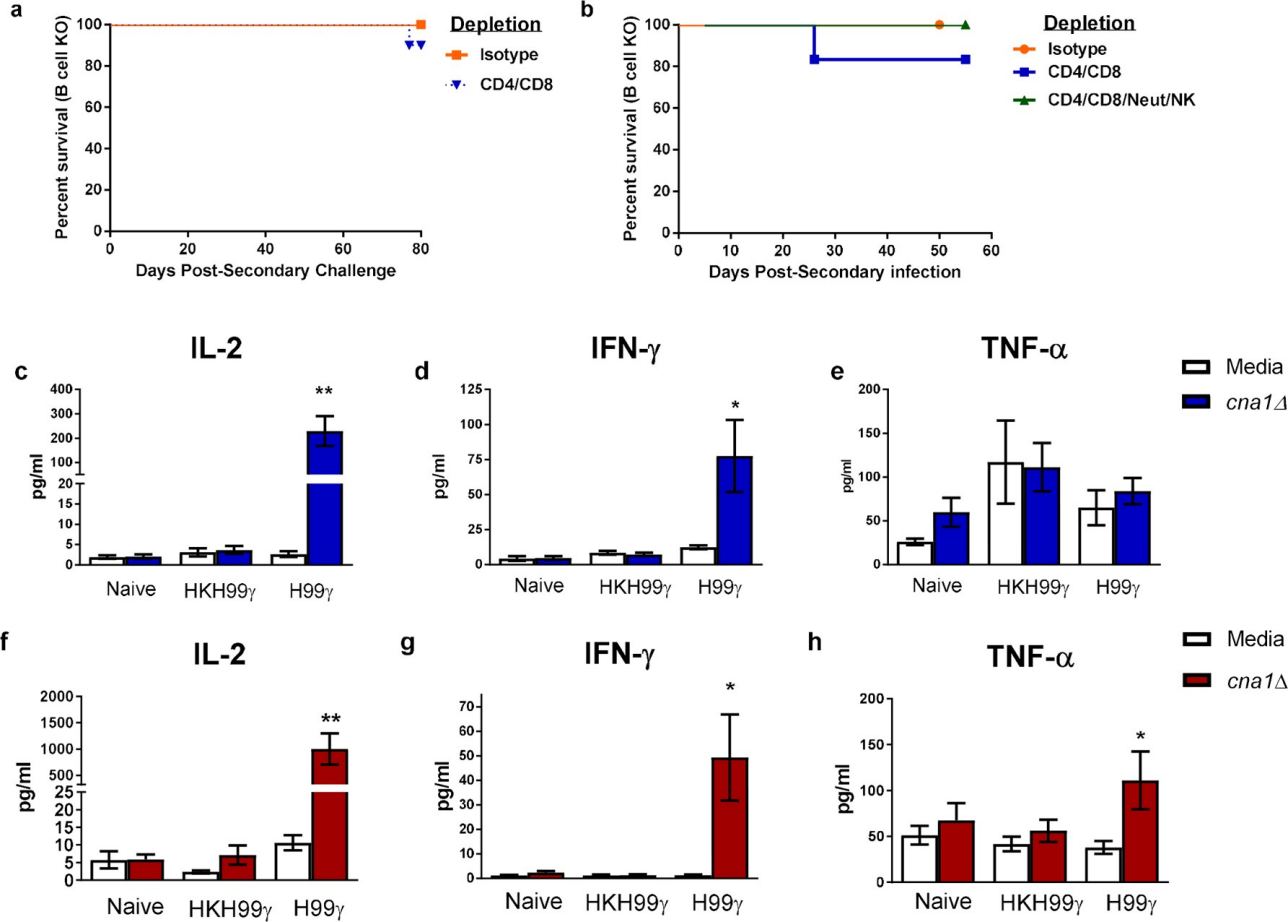
Macrophages from protectively immunized mice have enhanced cytokine recall responses when stimulated with *C*. *neoformans ex vivo*. (a-b) B cell knockout mice received an initial immunization with heat killed *C*. *neoformans* strain H99γ or *C*. *neoformans* strain H99γ and were allowed 70 days to resolve the infection. Mice were then treated with isotype control antibodies or depleted of CD4^+^/CD8^+^ T cells (a) and/or depleted of NK cells and neutrophils (b) prior to and during challenge with *C*. *neoformans* strain H99. Alternatively, macrophages were isolated from the lungs (c-e) and spleens (f-h) of the immunized mice, as well as from naïve mice, and cultured *ex vivo* with *C*. *neoformans* strain *cna1*Δ for 24 h. Supernatants were collected and analyzed for IL-2, IFN-γ, and TNF-α production. The survival data shown are from one experiment using 5–10 mice per experimental group. Cytokine recall data shown are means +/- SEM of 3–4 independent experiments using 20–25 mice per group. (*p < 0.05, **p < 0.01 compared to media within the immunization group).

Previous studies showed that mice given an experimental pulmonary infection with a fungal pathogen, *C*. *neoformans*, engineered to produce interferon-γ (IFN-γ), denoted H99γ, were protected against subsequent challenge with the fully pathogenic, non-IFN-γ producing *C*. *neoformans* strain H99 [15]. Protection was also observed in H99γ immunized mice deficient in B cells or depleted of CD4^+^ and/or CD8^+^ T cells and challenged with wild-type (WT) yeast [10, 11, 15]. To elucidate the effector cell population required for protection, B cell KO mice were protectively or non-protectively immunized with *C*. *neoformans* strain H99γ or heat killed *C*. *neoformans* strain H99γ (HKH99γ), respectively. The mice were rested for 70 days, depleted of both CD4^+^ and CD8^+^ T cells or given isotype control antibodies and then challenged with WT *C*. *neoformans* strain H99 (Fig 1A). Cell depletions were maintained throughout the observation period and were verified by flow cytometry (S1 Fig). B cell KO mice and B cell KO mice depleted of both T cell subsets showed a 90% (Fig 1A; *p* = 0.3173) and 80% (Fig 1B; p = 0.3613) survival rate, respectively. These findings led us to hypothesize that the effector cell population responsible for protection in B and T cell-deficient mice is a member of the innate immune system. To investigate this hypothesis, we depleted protectively immunized B cell KO mice of both subsets of T cells as well as natural killer (NK) cells and neutrophils and subsequently challenged the mice with WT cryptococci (Fig 1B; S1 Fig). Remarkably, the mice rendered deficient in adaptive immune cells in addition to these two innate cell populations were 100% protected against challenge (*p* = 1.0), demonstrating that neither T cells, B cells, neutrophils nor NK cells were necessary for protective immunity.

### Macrophages from protectively immunized mice have heightened cytokine recall responses

Previous studies by our lab and others have demonstrated that classically activated macrophages (M1) are important for protective immune responses against *C*. *neoformans* as they produce pro-inflammatory cytokines and can kill cryptococci via nitric oxide production [16–22]. Recent studies investigating innate memory-like responses have shown that trained monocytes/macrophages have elevated cytokine recall responses following secondary exposure to an antigen [8]. Therefore, we sought to investigate whether macrophages from protectively immunized mice were trained to respond quickly upon secondary exposure to *C*. *neoformans*. Macrophages were isolated from the lungs of mice that were immunized with *C*. *neoformans* strain H99γ or HKH99γ and rested for 70 days as well as naïve mice. The macrophages were cultured *ex vivo* for 24 hours with a *C*. *neoformans* strain H99 calcineurin subunit α deletion mutant (*cna1*Δ) which is unable to proliferate at 37°C and, thus, will not outgrow the macrophages in culture. The supernatants were then analyzed for the production of cytokines associated with pro-inflammatory and protective anti-cryptococcal responses. Interestingly, pulmonary macrophages from protectively immunized mice stimulated with cryptococci produced significantly more IL-2 (*p* = 0.0013) and IFN-γ (p = 0.0191), cytokines associated with protective responses (Fig 1C and 1D). No difference in TNF-α production was detected between stimulated versus unstimulated pulmonary macrophages from protectively immunized mice (Fig 1E).

As the spleen is a reservoir for immune cells including macrophages, and macrophages are known to traffic back to the spleen after the resolution of infection, we also tested splenic macrophages from immunized and naïve mice for cytokine recall abilities. A significant increase in IL-2 (*p* = 0.0035), IFN-γ (*p* = 0.0134) and TNF-α (*p* = 0.0289) production was detected by stimulated macrophages from protectively immunized mice compared to non-protectively immunized mice (Fig 1F–1H). No significant increase in cytokine production was detected in macrophages from naïve or HKH99γ immunized mice from both pulmonary and splenic macrophages (Fig 1C–1H). To ensure that the cytokines detected in culture were produced by macrophages, we analyzed the intracellular cytokine production of the macrophages by imaging flow cytometry. After 6 hours in culture, macrophages were confirmed positive for the macrophage markers CD11b and CD64. We did not further access F4/80 cell surface expression as anti-F4/80 antibodies still bound to the cells following positive selection are likely to sterically hinder binding of anti-F4/80 antibodies used for flow cytometry. The flow cytometry data confirmed that the CD11b^+^CD64^+^ macrophages did indeed produce the cytokines IL-2, IFN-γ, and TNF-α (S2 Fig).

**Fig 2:**
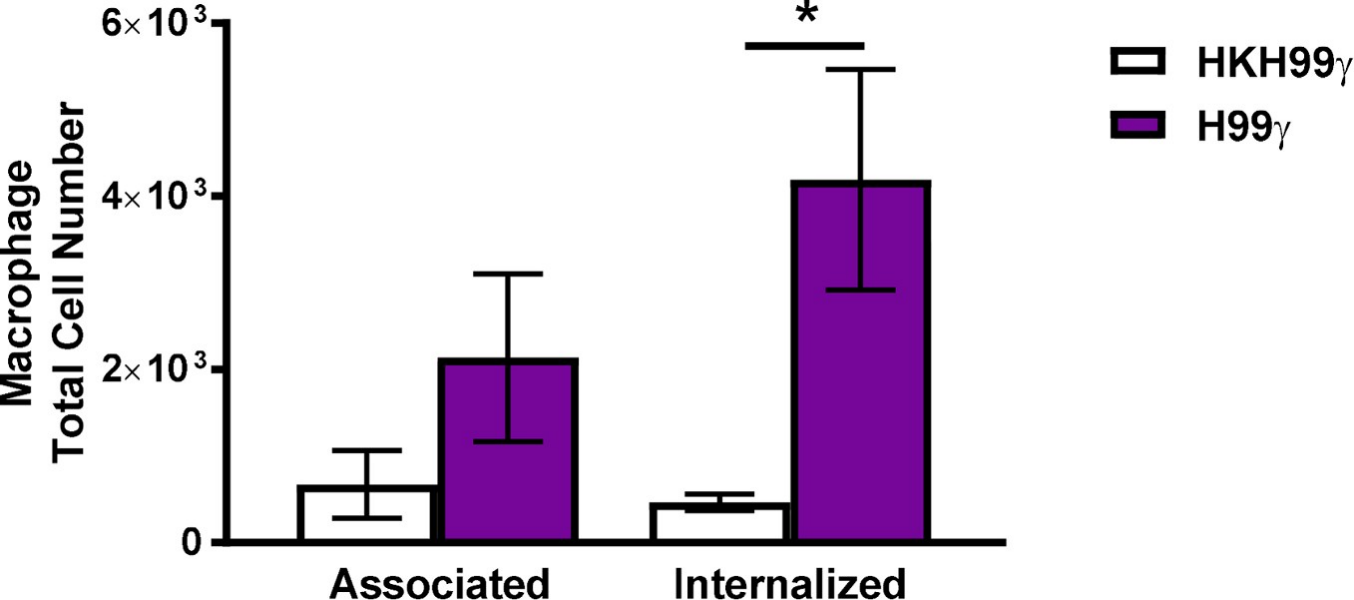
Macrophages from protectively immunized mice have increased cryptococcal phagocytic activity. BALB/c mice received an initial immunization with heat killed *C*. *neoformans* strain H99γ or *C*. *neoformans* strain H99γ and allowed to rest for 70 days. Macrophages were then isolated from the lungs of the immunized mice and cultured *ex vivo* with an mCherry expressing strain *C*. *neoformans* for 6 h at a 5:1 ratio (*C*. *neoformans* to macrophage ratio). Macrophage association with cryptococcal cells and internalization of cryptococcal cells was determined. Data shown are means +/- SEM of 3 independent experiments using 10 mice per group. (*p < 0.05).

Previous data from our lab indicated that macrophages from protectively immunized mice are more fungistatic than those from non-protectively immunized mice [19]. In the current study, we sought to determine if there were differences in phagocytic capabilities of the macrophages. Splenic macrophages isolated from HKH99γ immunized or H99γ immunized mice were cultured with an mCherry expressing strain of *C*. *neoformans* (KN99mCH) for 6 hours and subsequently analyzed for macrophage association with or internalization of the cryptococci as described previously [23]. We observed a shift in increased association of macrophages from protectively immunized mice with cryptococcal cells, though the difference was not significant (Fig 2). However, significantly more macrophages from H99γ immunized mice internalized cryptococci compared to macrophages derived from HKH99γ immunized mice. Altogether, these data suggest that immunization with the IFN-γ producing strain primes the macrophages to respond to a secondary exposure to the yeast both by increased internalization of the pathogen and increased proinflammatory cytokine production, which likely aids in protection during *in vivo* challenge.

### IFN-γ training of macrophages with *C*. *neoformans* H99γ is *Cryptococcus*-specific

**Fig 3:**
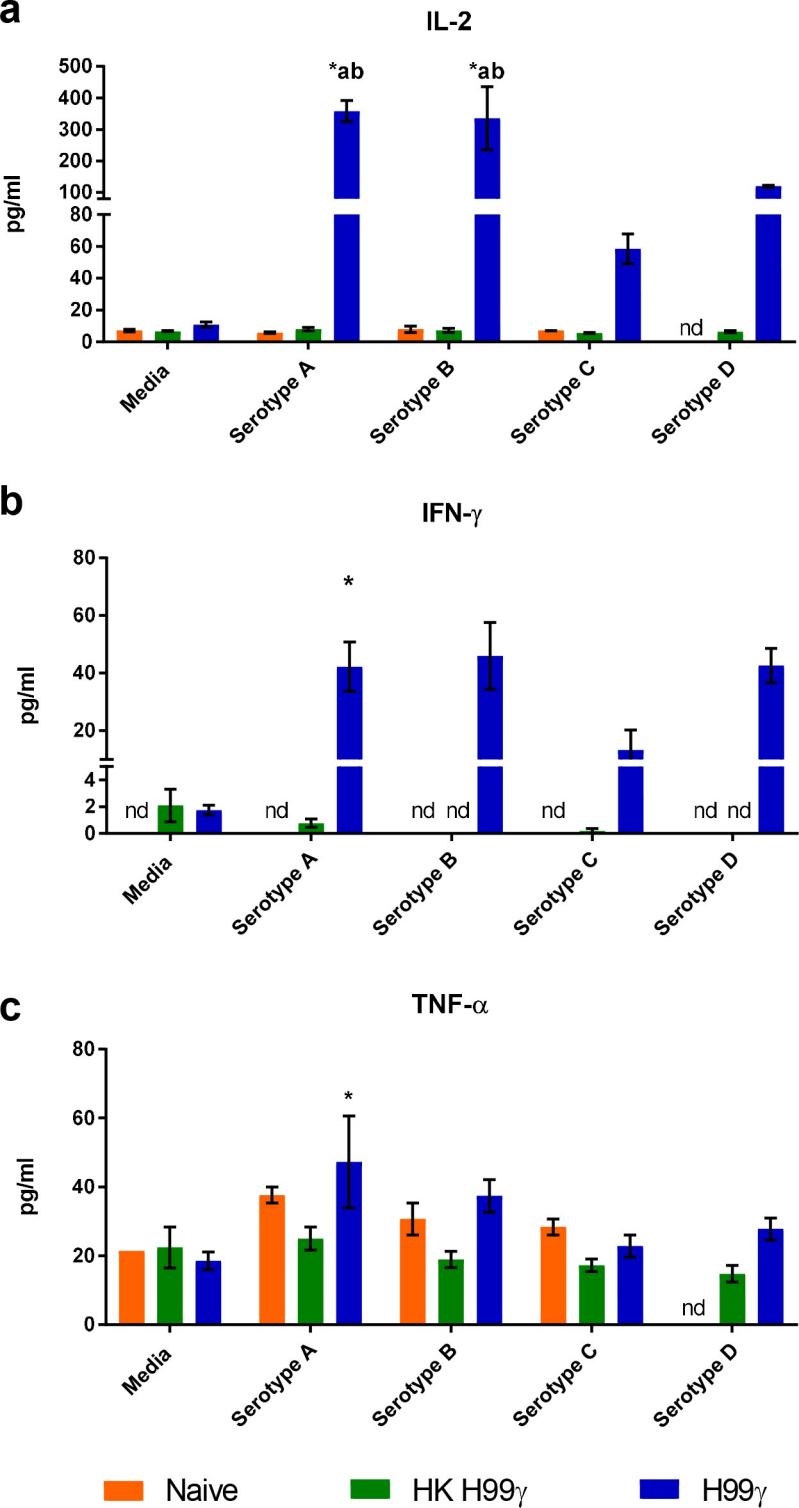
Macrophages from protectively immunized mice have enhanced cytokine production in response to disparate cryptococcal serotypes. BALB/c mice received an initial immunization with heat killed *C*. *neoformans* strain H99γ or *C*. *neoformans* strain H99γ and allowed to rest for 70 days. Macrophages were then isolated from the spleens of the immunized mice, as well as naïve mice, and cultured *ex vivo* with heat killed *C*. *neoformans* H99, *C*. *deuterogattii* R265, *C*. *bacillisporus* WSA87, *C*. *deneoformans* 52D or media alone for 24 h. Supernatants were collected and analyzed for IL-2 (a), IFN-γ (b) and TNF-α (c) production. Data shown are means +/- SEM of 2 independent experiments using 10 mice per group. A two-way ANOVA with Tukey’s multiple comparison test was performed to compare cytokine production from each stimulus within one immunization group to media alone as well as to compare cytokine production elicited by each stimulus between the 3 immunization groups. (* p < 0.05 compared to media within the same immunization group; a, p < 0.05 compared to naïve, b, p < 0.05 compared to HKH99γ within each secondary stimulation group; nd, not detected).

Cytokine recall responses of pulmonary and splenic macrophages from protectively immunized mice to *C*. *neoformans* appeared to follow a similar trend. Next, we sought to assess the specificity of the cytokine recall response to other members of the *Cryptococcus* species complex. Previous studies have shown the importance of T cell responses to protect against disparate serotypes *in vivo* [24], however, evaluation of the macrophage contribution to protection is unknown. Considering that significantly more macrophages can be obtained from splenic tissues than lung, we elected to use splenic macrophages to significantly reduce the number of mice needed to perform subsequent assays. We cultured macrophages from HKH99γ immunized or H99γ immunized mice for 24 hours with heat killed *C*. *neoformans* strain H99 (serotype A), *C*. *deuterogattii* strain R265 (serotype B), *C*. *bacillisporus* strain WSA87 (serotype C), *C*. *deneoformans* strain 52D (serotype D) or media alone and measured the cytokines present in the supernatants. Macrophages from protectively immunized mice produced significantly more IL-2 when stimulated with serotypes A, B, and D compared to stimulated macrophages from naïve and non-protectively immunized mice (Fig 3A). The macrophages from protectively immunized mice also produced significantly more IL-2 when stimulated with serotypes A, B, and D and more IFN-γ and TNF-α when stimulated with serotype A compared to unstimulated cells (Fig 3A–3C). For IFN-γ, there was a trend towards increased cytokine production in macrophages from H99γ immunized mice stimulated with all four serotypes, however it was not statistically significantly increased compared to either unstimulated cells or stimulated cells from naïve or HKH99γ immunized mice. Nevertheless, the lack of statistical significance observed is likely due to the lack of IFN-γ production by macrophages from naïve or non-protectively immunized mice following stimulation with the various serotypes, which prevented the performance of statistical analysis. Interestingly, the levels of TNF-α was relatively low, with the exception of macrophages from protectively immunized mice stimulated with serotype D. Overall, there was no significant change in TNF-α secretion measured across all conditions except for macrophages from H99γ immunized mice stimulated with serotypes A and D compared to unstimulated cells and compared to serotype D stimulated cells from naïve and HKH99γ immunized mice (Fig 3C). Previous studies show that immunization of mice with heat killed *C*. *neoformans* strains (including HKH99γ) or live *C*. *deneoformans* 52D does not result in protective immunity [11, 15, 24]. However, these data suggest that immunization with H99γ stimulates macrophages to provoke a memory-like response following stimulation with other *Cryptococcus* serotypes.

**Fig 4:**
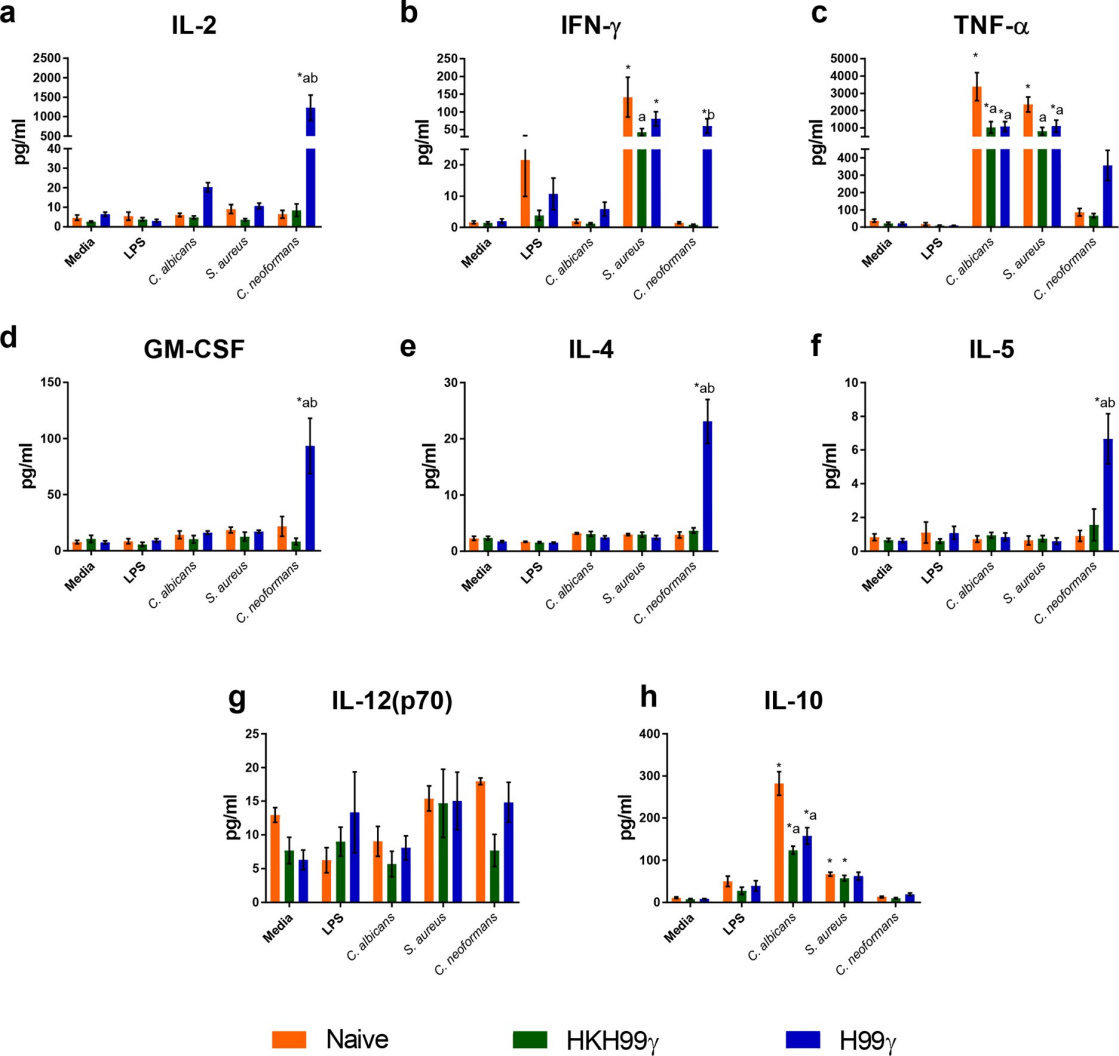
Cytokine recall responses from protectively immunized mice are cryptococcal specific. BALB/c mice received an initial immunization with heat killed *C*. *neoformans* strain H99γ or *C*. *neoformans* strain H99γ and allowed to rest for 60 days. Macrophages were then isolated from the spleens of the immunized mice, as well as from naïve mice, and cultured *ex vivo* with LPS, heat killed *Candida albicans*, heat killed *Staphylococcus aureus* or *C*. *neoformans* strain *cna1*Δ for 24 h. Supernatants were collected and analyzed for IL-2 (a), IFN-γ (b) and TNF-α (c), GM-CSF (d), IL-4 (e), IL-5 (f), IL-12(p70) (g), or IL-10 (h) production. Data shown are means +/- SEM of 3 independent experiments using 20–25 mice per group. A two-way ANOVA with Tukey’s multiple comparison test was performed to compare cytokine production from each stimulus within one immunization group to media alone as well as to compare cytokine production elicited by each stimulus between the 3 immunization groups. (* p < 0.05 compared to media within the same immunization group; a, p < 0.05 compared to naïve, b, p < 0.05 compared to HKH99γ within each secondary stimulation group).

Our next question was whether or not immunization with *C*. *neoformans* H99γ can induce memory-like responses against non-cryptococcal antigens. To answer this question, we cultured splenic macrophages from immunized and naïve mice for 24 hours with LPS, heat killed *Candida albicans*, heat killed *Staphylococcus aureus* or *C*. *neoformans cna1*Δ to access the specificity of the cytokine recall response. Cytokine levels in cultures containing macrophages from protectively immunized mice appeared to only produce significantly elevated levels of several cytokines upon stimulation with *C*. *neoformans*. Macrophages from protectively immunized mice exposed to *C*. *neoformans* secreted IL-2 at significantly elevated levels compared to macrophages cultured in media alone or macrophages from naïve and non-protectively immunized mice (Fig 4A). Comparable levels of IFN-γ were secreted by macrophages from all immunization groups when stimulated with *S*. *aureus*, however, only the macrophages from the protectively immunized mice produced significant levels of IFN-γ in response to *C*. *neoformans* (Fig 4B). Likewise, TNF-α was detected in supernatants of all immunization groups during co-culture with *S*. *aureus* and *C*. *albicans* at comparable levels suggesting no trained response to these microbes. However, TNF-α production was increased by 4-fold in protectively immunized mice compared to all other groups following cryptococcal stimulation, although not statistically significant (Fig 4C). Granulocyte macrophage-colony stimulating factor (GM-CSF) and Th2-associated cytokines IL-4 and IL-5 were only significantly increased in macrophages from the protectively immunized mice co-cultured with *Cryptococcus*, indicating that these responses were also *Cryptococcus*-specific (Fig 4D–4F). We do note that the IL-5 levels observed were relatively low (<8 pg/ml), and likely not biologically relevant. Similarly, the levels of IL-12p70 were low and did not appear to show bias to any immunization group or stimuli (Fig 4G). Interestingly, macrophages from all groups produced elevated levels of the immune regulatory cytokine IL-10 when stimulated with *C*. *albicans* (123–282 pg/ml) or *S*. *aureus* (57–67 pg/ml; Fig 2H). However, IL-10 was not produced in response to *C*. *neoformans* stimulation in any immunization group (Fig 4H). Overall, these data suggest that macrophages from protectively immunized mice have a significant *C*. *neoformans*-specific cytokine recall response and are in contrast with previous reports demonstrating a lack of specificity in the macrophages/monocytes trained with *C*. *albicans* or β-glucan [8]. Thus, an alternative mechanism may be responsible for the induction of enhanced cytokine recall responses by macrophages from H99γ immunized mice.

**Fig 5:**
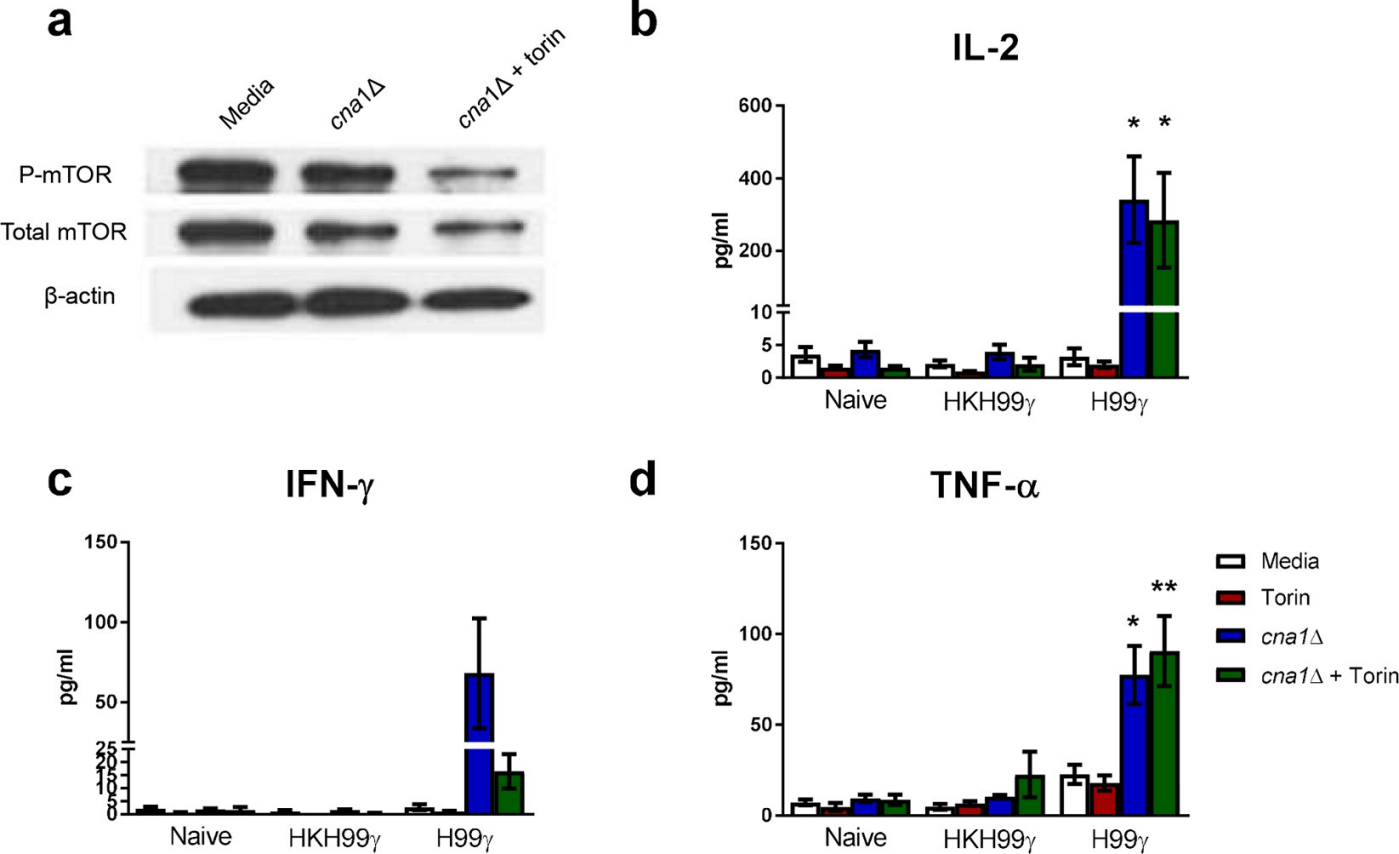
Cytokine recall responses from IFN-γ primed macrophages do not require mTOR activation. BALB/c mice received an initial immunization with heat killed *C*. *neoformans* strain H99γ or *C*. *neoformans* strain H99γ and allowed to rest for 60 days. Macrophages were then isolated from the spleens of the immunized mice, as well as from naïve mice, and cultured *ex vivo* with *C*. *neoformans* strain *cna1*Δ +/- torin for 24 h. Protein was extracted from the macrophages and analyzed for total mTOR and phosphorylated mTOR (a). Supernatants were collected and analyzed for IL-2 (b), IFN-γ (c) and TNF-α (d) production. Data shown are means +/- SEM of 3 independent experiments using 10–15 mice per group. Protein expression is representative of 3 independent experiments. (*p < 0.05, **p < 0.01 compared to media within the same immunization group).

Previous studies have demonstrated that inhibition of mTOR results in a loss of β-glucan induced trained immunity in monocytes [9]. We, therefore, determined the outcome of mTOR inhibition on cytokine recall responses by macrophages isolated from protectively immunized mice following exposure with *C*. *neoformans*. We observed similar levels of total and phosphorylated mTOR (p-mTOR) within macrophages from protectively immunized mice following exposure to *Cryptococcus in vitro* compared to p-mTOR levels in unstimulated macrophages (Fig 5A). Treatment of macrophages from protectively immunized mice with torin, an ATP-competitive inhibitor of mTOR kinase activity, concurrent with *C*. *neoformans* stimulation resulted in a decrease in p-mTOR below baseline levels observed in unstimulated macrophages. However, torin treatment did not suppress production of IL-2 or TNF-α by macrophages from protectively immunized mice (Fig 5B and 5D). While not statistically significant, lower levels of IFN-γ production were observed in macrophages from protectively immunized mice in the presence of torin which may have a biological impact on subsequent macrophage recall responses (Fig 5C). These data suggest that the cytokine recall responses of IFN-γ primed macrophages is independent of the mTOR pathway and is induced by a mechanism distinct from that observed for β-glucan or *C*. *albicans* induced trained immunity.

### IFN-γ immune primed macrophages activate the STAT1 pathway during secondary challenge

**Fig 6:**
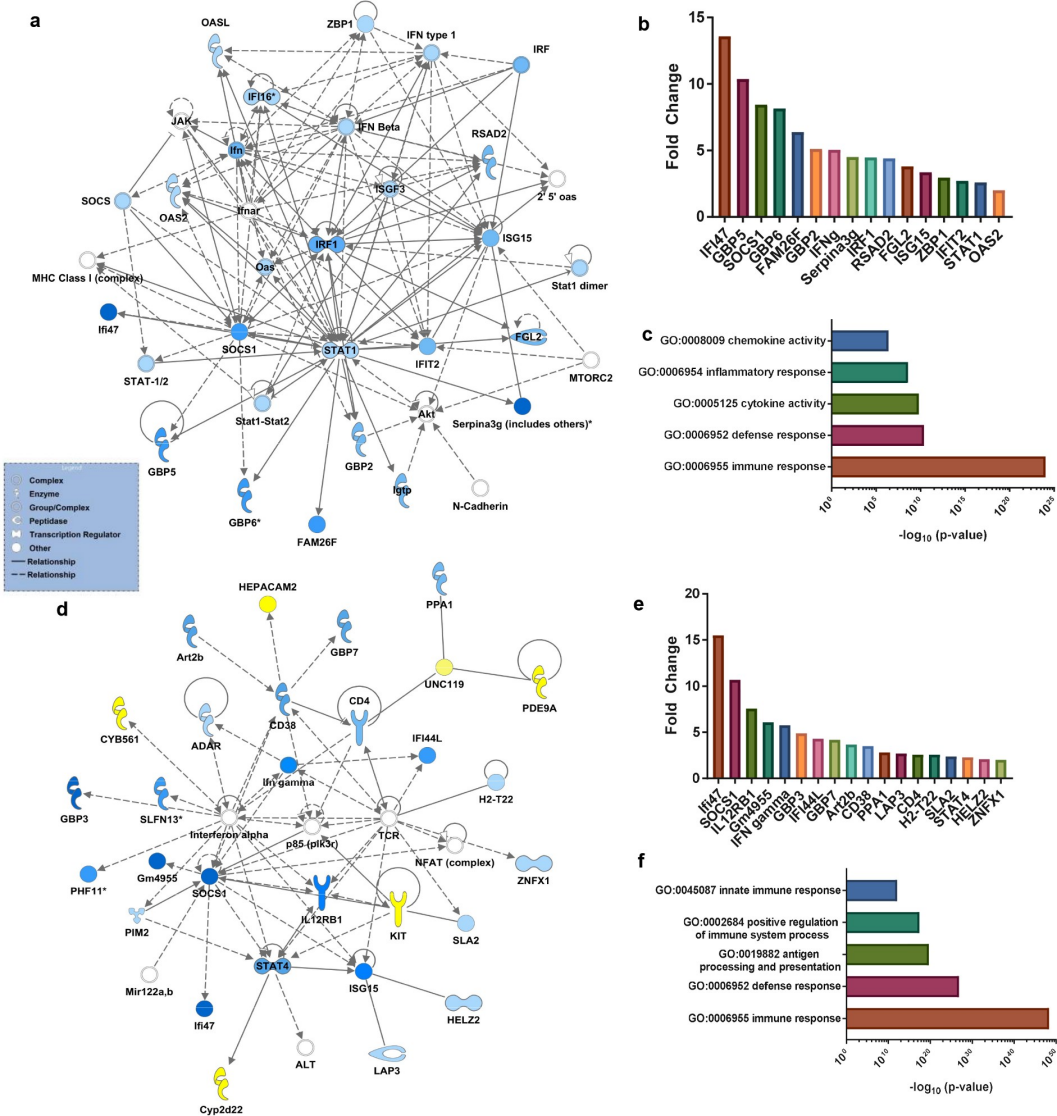
Proinflammatory networks are rapidly up-regulated in pulmonary macrophages of protectively immunized mice following challenge with *C*. *neoformans*. BALB/c mice received an immunization with heat killed *C*. *neoformans* strain H99γ or *C*. *neoformans* strain H99γ and allowed to rest for 60 days. Mice were then challenged with WT *C*. *neoformans* strain H99 and subsequently sacrificed at day 1 or day 3 post-challenge and total RNA was extracted and sequenced from isolated pulmonary macrophages. Top canonical pathways and networks were predicted by the Ingenuity Pathway Analysis software. Top networks up-regulated in pulmonary macrophages from protectively immunized mice compared to non-protectively immunized mice as ranked by p-value (day 1, a; day 3, d). Blue and yellow represent genes up-regulated or down-regulated, respectively, in macrophages from protectively immunized compared to non-protectively immunized mice. Fold change values for up-regulated genes represented in networks (day 1, b; day 3, e). Top 5 immune response related gene ontology (GO) terms ranked by p value (day 1, c; day 3, f). Data were generated from a merged data set from 3 independent experiments with 20–25 mice per group.

We performed whole transcriptome analysis of macrophages isolated from protectively compared to non-protectively immunized mice at days 1 and 3 post-challenge with WT *C*. *neoformans* yeast as well as macrophages from immunized but unchallenged mice. Ingenuity Pathway Analysis (IPA) of RNA-Seq results showed a rapid up-regulation of canonical pathways for interferon and IL-17 signaling in macrophages from protectively immunized mice compared to non-protectively immunized mice at day one post-challenge (Table 1). Notably, transcripts for *STAT1*, *SOCS1*, *IFIT2*, *NOS2*, *IFNG*, *CXCL10*, and *IL6* are among the genes up-regulated in the macrophages from protectively immunized mice at day-one post-challenge. Also, network analysis showed an up-regulation of the STAT1 network in macrophages from the protectively immunized mice (Fig 6A and 6B), confirming our previous findings [19]. Gene ontology (GO) analysis of RNA-Seq results from macrophages isolated at day 1 post-challenge resulted in GO terms associated with pro-inflammatory and anti-microbial pathways (Fig 6C; S1 Table). IPA of RNA-Seq results from macrophages isolated at day 3 post-challenge showed a rapid up-regulation of canonical pathways associated with T and B cell signaling, communication between the innate and adaptive immune system, and overall cytokine responses (Table 1). IPA also revealed up-regulation of networks associated with protective immunity and M1 macrophage activation as transcripts for genes including *SOCS1*, *STAT4*, *IL12B1*, and *IFNG* were up-regulated in the primed macrophages (Fig 6D and 6E). GO analysis of RNA-Seq results from macrophages isolated at day 3 post-challenge revealed up-regulation of pathways associated with a protective immune response in macrophages from protectively immunized mice (Fig 6F; S2 Table). Analysis of genes from immunized, unchallenged mice did not reveal significant changes in immune related genes with the exception of VCAM1 (2.18 fold increase) and CXCL9 (3.01 fold increase) in protectively immunized compared to non-protectively immunized mice. Altogether, these data demonstrate that immunization with *C*. *neoformans* strain H99γ primes the anti-microbial response of macrophages *in vivo* to secondary exposure to the yeast.

**Table 1:** IPA canonical pathways upregulated in pulmonary macrophages in protectively immunized mice.

**Day 1 Post-Challenge—Canonical Pathway**	**p-value**	**Molecules**
Activation of IRF by Cytosolic Pattern Recognition Receptors	7.32E-09	LTA, ZBP1, IKBKE, IL6, IFIT2, STAT1, ISG15
Interferon Signaling	8.06E-09	IFNG, SOCS1, STAT1, TAP1, IRF1, ISG15
IL-17 Signaling	2.12E-08	CXCL10, CXCL11, CCL2, TIMP1, IL6, PTGS2, NOS2
Type I Diabetes Mellitus Signaling	2.16E-08	IFNG, SOCS3, SOCS1, LTA, IKBKE, NOS2, STAT1, IRF1
Role of Hypercytokinemia/ hyperchemokinemia in the Pathogenesis of Influenza	2.47E-08	CXCL10, IFNG, CCR5, IL36G, CCL2, IL6
**Day 3 Post-Challenge—Canonical Pathway**	**p-value**	**Molecules**
Altered T Cell and B Cell Signaling in Rheumatoid Arthritis	1.34E-13	IFNG, IL12A, SPP1, HLA-DQA1, IL22, HLA-DQB1, TLR9, L17A, Tlr12, IL18, IL36G, CD40, CXCL13, CSF1, PRTN3, H2-Eb2, LTA,HLA-DMB, TLR1, HLA-DRA, CSF2, HLA-DRB5
Communication between Innate and Adaptive Immune Cells	2.07E-12	B2M, IFNG, IL12A, HLA-4, CD4, CCL5, IGHG1, TLR9, CD8A, CD8B, Tlr12, CXCL10, HLA-G, IL18, IL36G, CD40, TLR1, HLA-DRA, CSF2, HLA-E, HLA-DRB5
Dendritic Cell Maturation	2.36E-12	B2M, IL12A, HLA-A, LEPR, NFKBIE, HLA-DQA1, HLA-DQB1, IGHG1, JAK2, FCGR2B, FCGR1A, CD1D, IL36G, HLA-DMB, HLA-DRA, STAT1, FCGR3A/FCGR3B, IKBKE, TLR9, STAT4, IL18, Cd1d2, CD40, H2-Eb2, LTA, STAT2, IRF8, CSF2, HLA-DRB5
Antigen Presentation Pathway	7.27E-12	B2M, IFNG, PSMB9, HLA-A, HLA-DQA1, PSMB8, CD74, TAP1, HLA-G, HLA-DMB, HLA-DRA, TAP2, HLA-DRB5, HLA-E
Type I Diabetes Mellitus Signaling	2.01E-11	IFNG, SOCS3, SOCS1, IL12A, ICA1, GZMB, HLA-A, NFKBIE, HLA-DQA1, IKBKE, JAK2, HLA-DQB1, IRF1, HLA-G, H2-Eb2, LTA, HLA-DMB, HLA-DRA, NOS2, STAT1, HLA-E, HLA-DRB5
T Helper Cell Differentiation	2.25E-11	BCL6, CD28, CD40, CD40LG, CD80, CD86, CXCR5, FOXP3, GATA3, ICOS, ICOSLG/LOC102723996, Ifn gamma receptor, IFNG, IL-4 receptor, IL10, IL10R, IL12 (family), IL12 receptor, IL12RB2, IL13, IL17A, IL17F, IL18, IL18R1, IL2, IL21, IL21R, IL23R, IL2RA, IL4, IL5, IL6, IL6 receptor, IL6ST, MHC Class II (complex), RORC, STAT1, STAT3, STAT4, STAT6, TBX21, TCR, Tgf beta receptor, TGFB1, TNF, TNF receptor, tretinoin
Interferon Signaling	8.26E-11	BAK1, BAX, BCL2, glucocorticoid, IFI35, IFI6, IFIT1, IFIT3, IFITM1, IFITM2, IFITM3, IFN alpha/beta, IFNAR1, IFNAR2, IFNG, IFNGR1, IFNGR2, IRF1, IRF9, ISG15, ISGF3, JAK1, JAK2, MED14, MX1, OAS1, PIAS1, PSMB8, PTPN2, RELA, SOCS1, STAT1, Stat1 dimer, Stat1-Stat2, STAT2, TAP1, TYK2

Ingenuity Pathway Analysis (IPA) showing canonical pathways most upregulated in pulmonary macrophages from *C*. *neoformans* strain H99γ compared to heat killed *C*. *neoformans* strain H99γ immunized mice, ranked by p-value. Data were generated from a merged data set of 3 independent experiments, 20–25 mice per group.

### Cytokine recall responses in macrophages from *C*. *neoformans* H99γ immunized mice require epigenetic modifications

**Fig 7:**
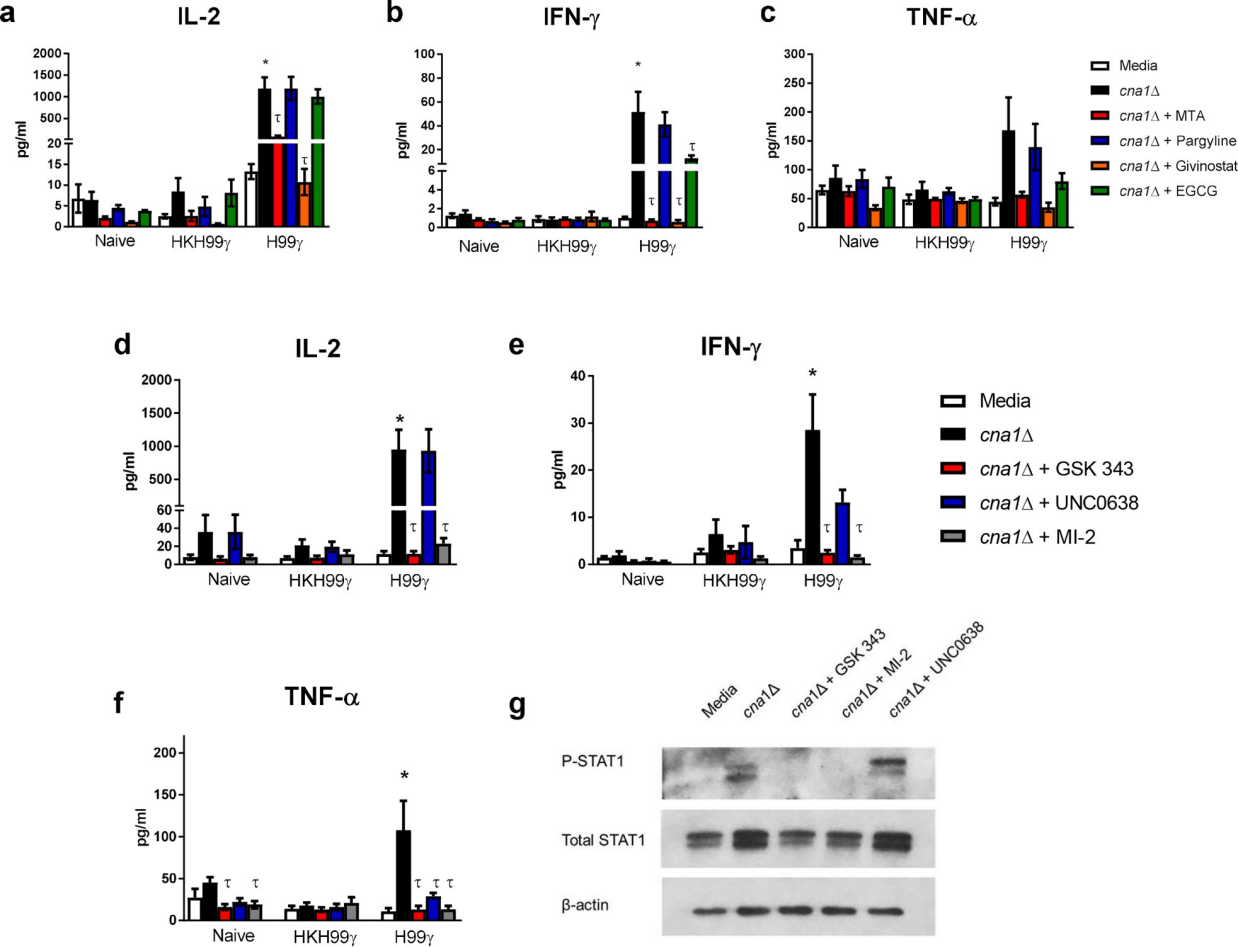
Cytokine recall responses in macrophages from *C*. *neoformans* H99γ immunized mice require epigenetic modifications. BALB/c mice received an initial immunization with heat killed *C*. *neoformans* strain H99γ or *C*. *neoformans* strain H99γ and allowed to rest for 60 days. Macrophages were then isolated from the spleens of the immunized mice, as well as from naïve mice, and cultured *ex vivo* with *C*. *neoformans* strain *cna1*Δ +/- MTA, pargyline hydrochloride, givinostat or EGCG for 24 h (a-c) or *C*. *neoformans* strain *cna1*Δ +/- GSK343, UNC 0638 or MI-2 for 24 h (d-g). Supernatants were collected and analyzed for IL-2 (a, d), IFN-γ (b, e) and TNF-α (c, f) production. Alternatively, macrophages were collected after the 24h period and analyzed for total and phosphorylated STAT1 (g). Data shown are means +/- SEM of 3 independent experiments using 20–25 mice per group. Protein expression is representative of 3 independent experiments. (*p < 0.05, compared to media within the same immunization group; τ < 0.05 compared to *cna1*Δ within the same immunization group).

Studies show that β-glucan training of monocytes induces histone modifications related to genes associated with protective immunity against various microbial pathogens [4, 7, 8, 25]. To investigate how IFN-γ priming results in the anti-cryptococcal activity of the macrophages, we cultured splenic macrophages from protectively and non-protectively immunized mice or naïve mice with 5′-deoxy-5′-methylthioadenosine (MTA), a histone methyltransferase (HMT) inhibitor, pargyline, a histone demethylase (HDM) inhibitor, givinostat, a histone acetyltransferase (HDAC) inhibitor, or epigallocatechin gallate (EGCG), a histone acetyltransferase (HAT) inhibitor. As shown in Fig 1, stimulation of the macrophages with *cna1*Δ induced elevated cytokine production only in macrophages from protectively immunized mice. Interestingly, co-culture with the HMT inhibitor MTA and HDAC inhibitor givinostat significantly decreased IL-2 and IFN-γ production by macrophages from protectively immunized mice (Fig 7A and 7B). In addition, EGCG reduced IFN-γ production by macrophages from protectively immunized mice (Fig 7B). TNF-α production showed a similar trend to IFN-γ, however the values were not statistically significant (Fig 7C). Overall, these data suggest that the increased cytokine recall potential of macrophages from protectively immunized mice is due to changes in histone modifications elicited by immunization with *C*. *neoformans* strain H99γ.

We subsequently focused on modifications of methylation patterns on histones in macrophages. We analyzed cytokine recall responses by splenic macrophages from naïve, non-protectively immunized and protectively immunized mice when cultured with inhibitors for enzymes that induce specific methylation patterns on histones. The EZH2 inhibitor GSK343 prevents tri-methylation of histone 3 lysine 27 (H3K27me3) which is associated with repression of transcription. UNC0638, an inhibitor of G9a, prevents the repressive modification H3K9me3. Finally, we used an inhibitor of the MLL complex, MI-2, thus preventing addition of H3K4me3 which is associated with active transcription. As expected, inhibition of H3K4me3 resulted in a decrease in IL-2 (p < 0.05), IFN-γ (p < 0.01) and TNF-α (p < 0.01) cytokine production by *in vivo* IFN-γ primed macrophages (Fig 7D–7F). In addition, inhibition of the repressive mark H3K27me3 also resulted in decreased IL-2 (p < 0.05), IFN-γ (p < 0.01) and TNF-α (p < 0.01) cytokine production. This may indicate an indirect role for H3K27me3 that aids in cytokine production by the trained macrophages. Inhibition of H3K9me3 resulted in a decrease in IFN-γ, although not significant (p > 0.05), and TNF-α (p < 0.05) cytokine production, but not IL-2 production by IFN-γ trained macrophages.

IFN-γ immune primed macrophages rapidly up-regulate the STAT1 pathway following challenge with *C*. *neoformans in vivo*. Consequently, we examined the effects of the methylation inhibitors on STAT1 activation. Stimulation of splenic macrophages from protectively immunized mice with *C*. *neoformans* increases total STAT1 and phosphorylated STAT1 (pSTAT1) compared to unstimulated macrophages (Fig 7G). Interestingly, co-culture of the macrophages with *C*. *neoformans* and GSK343 or MI-2 resulted in decreased expression of total STAT1 protein and pSTAT1 (Fig 7G), suggesting a role for H3K27me3 and H3K4me3 in STAT1 pathway activation. Inhibition of H3K9me3, however, had no effect on total STAT1 or pSTAT1 expression. Altogether, these data suggest that changes in the methylation patterns contribute to the cytokine recall responses and activation of the STAT1 pathway in macrophages from protectively immunized mice.

### *In vivo* training of macrophages with IFN-γ results in lasting STAT1 binding to promotor regions of known IFN-induced genes

Data from the previous assays show the importance of STAT1 signaling to the enhanced cytokine production observed in macrophages from protectively immunized mice. We next sought to determine if the immunization with *C*. *neoformans* strain H99γ led to an alteration in STAT1 binding at promotor regions of certain known IFN-γ-induced genes. For these experiments, mice were immunized with either HKH99γ or H99γ and allowed to rest for 70 days. Macrophages were then isolated from the lungs of the immunized mice and chromatin immunoprecipitation (ChIP) was performed using an antibody against STAT1. The resulting DNA was amplified by real-time ChIP qPCR using a custom ChIP array. Target genes were chosen based on the results of the RNA-seq analysis on day 1 post-challenge, selecting genes that were significantly up-regulated in macrophages from H99γ immunized mice compared to macrophages from HKH99γ immunized mice. The data revealed an overall trend for increased percent IP fold enrichment of STAT1 binding to sites 1kb upstream of the transcription start site for the selected genes in macrophages from protectively immunized mice compared to non-protectively immunized mice (Table 2). We observed significantly increased binding of STAT1 to promotor regions of guanylate binding protein 6 (*GBP6*), *CXCL10*, and *CIITA*. We also detected increased binding of STAT1 to promotor regions of *IRF-1*, *CXCL11*, and *IFIT2* with p-values approaching statistical significance. Again, transcripts for each of these genes are significantly elevated post-challenge suggesting that immunization with H99γ induced lasting STAT1 binding at promotor regions of select IFN-induced genes within macrophages of protectively immunized mice thus priming them to readily respond to a second *Cryptococcus* challenge.

**Table 2:** STAT1 IP fold enrichment in pulmonary macrophages.

Gene	HKH99γ	H99γ	p value
**GBP6**	**0.90 ± 0.42**	3.57 ± 0.15**	**0.002**
**CXCL10**	**1.17 ± 0.67**	3.27 ± 0.35**	**0.008**
**CIITA**	**1.50 ± 0.85**	2.90 ± 1.15*	**0.043**
IFIT2	1.10 ± 0.46	3.63 ± 1.69	0.067
CXCL11	1.60 ± 0.70	3.93 ± 1.55	0.076
IRF-1	0.97 ± 0.57	2.57 ± 1.03	0.078
iNOS	1.73 ± 1.08	3.53 ± 1.04	0.106
SOCS1	1.40 ± 0.85	3.53 ± .035	0.166
CXCL9	1.73 ± 0.71	3.80 ± 2.10	0.181
GBP2	1.57 ± 1.42	3.43 ± 1.56	0.200
IFI47	1.87 ± 0.61	2.6 ± 0.28	0.223
GBP5	1.47 ± 0.91	2.83 ± 1.46	0.241

STAT1 transcription factor binding at promotor regions of IFN-γ induced genes from pulmonary macrophages isolated from immunized mice, ranked by p-value. Data were generated from 3 independent experiments, 10 mice per group. (*p < 0.05, **p < 0.01)

GBP, guanylate binding protein; CXCL, chemokine (C-X-C- motif) ligand; CIITA, class II major histocompatibility complex transactivator; IFIT, interferon induced protein with tetratricopeptide repeats; IRF, interferon regulatory factor; iNOS, inducible nitric oxide synthase; SOCS, suppressor of cytokine signaling; IFI, interferon-γ inducible protein.

## Discussion

In the present study, we demonstrate that macrophages from mice immunized with an IFN-γ producing strain of *C*. *neoformans* are primed to rapidly respond to a subsequent exposure to this pathogen. The effect appears to be STAT1-dependent, as demonstrated by expedited transcription of genes that are directly and indirectly associated with the STAT1 pathway as well as increased binding of STAT1 at promotor regions of select genes. Activation of STAT1 in macrophages results in their polarization toward an M1 phenotype which has been widely demonstrated as anti-cryptococcal and necessary for protective immunity against *C*. *neoformans* [16, 17, 19–22, 26]. To date, myelofibrosis treatment of two patients with Ruxolitinib, which inhibits JAK1,2 and likely STAT1, is hypothesized to have contributed to the development of cryptococcosis in these individuals [27, 28].

Macrophages primed with IFN-γ respond quickly to a secondary signal, resulting in tumoricidal and microbicidal activities of these cells [29–37]. It seems likely that by immunizing mice with the IFN-γ producing strain of *C*. *neoformans*, the macrophages are primed *in vivo* to respond to a subsequent exposure to cryptococci. Remarkably, these macrophages remain trained at least as long as 70 days post-immunization. At this point, we are unable to ascertain if the macrophages in the lungs at day 70 post-immunization are the same macrophages present during immunization. Alveolar macrophages are known to self-renew [38], therefore some macrophage turnover is expected. Future studies to address the question of macrophage turnover and long-term training are required.

Trained immunity refers to a non-specific response of innate immune cells that is independent of T and B cells and increases resistance against reinfection [4–7]. Studies show that β-glucan, a major component of fungal cell walls, trains monocytes to respond with enhanced production of IL-6 and TNF-α not only when stimulated with *C*. *albicans*, but also following stimulation with LPS, *Mycobacterium tuberculosis*, and the TLR-2 agonist Pam3Cys [8] which differs from our model which suggests the *C*. *neoformans*/IFN-γ priming is specific for *C*. *neoformans*. A recent study investigating β-glucan training concluded that the trained monocytes displayed enhanced survival *in vitro* via partial inhibition of apoptosis and that the increased survival of these trained cells explained the elevated levels of pro-inflammatory cytokines elicited by LPS challenge compared to untrained cells [39]. This study also showed that systemic administration of β-glucan to mice rendered the animals more responsive to LPS challenge after 4 days, however the effect was short lived as the enhanced cytokine responses were lost by day 20 post-β-glucan immunization [39]. These results differ with those in our model in which animals are still protected and macrophages exhibit heightened cytokine responses as far as 70 days post-IFN-γ priming *in vivo*.

Immunization with the IFN-γ producing *C*. *neoformans* strain requires the STAT1 pathway to induce protection [16, 17], thus, the mechanisms for eliciting IFN-γ innate memory and β-glucan training are likely different. Training with β-glucan signals through the C-type lectin receptor Dectin-1 [8] which specifically recognizes β-glucan [40]. However, *C*. *neoformans* possesses a polysaccharide capsule that efficiently masks detection of several cell wall components, including β-glucan. Thus, it is unlikely that β-glucan in the *C*. *neoformans* cell wall would trigger innate memory in the macrophages. In addition, we demonstrate that the memory-like response of macrophages from protectively immunized mice does not require mTOR activity during the anamnestic response; a signaling pathway required for β-glucan induced training [9]. In fact, cytokine production increases when mTOR activation is inhibited. Recent studies have shown that IFN-γ priming can inhibit mTORC1 (of which mTOR is the catalytic subunit) in human macrophages, increasing pro-inflammatory cytokine production by translational suppression of repressors of inflammation [41]. A recent study using *M*. *bovis* Bacillus Calmette-Guerin (BCG) to induce memory-like innate immune priming in mice revealed that the BCG bacteria can infiltrate the bone marrow and prime the hematopoietic stem cells (HSCs) to generate monocytes/macrophages that are more antimycobacterial than controls [42]. This priming appears to be dependent on IFN-γ signaling and STAT transcription factors [42], while conversely, training of HSCs with β-glucan occurs via IL-1β and activation of the GM-CSF/CD131 axis [43]. These data indicate that there are disparate mechanisms that can lead to innate training in mice. In addition, defects in trained immunity have been documented in patients with STAT1-mediated chronic mucocutaneous candidiasis (CMC) and following blockade of IFN-γ in PBMCs of healthy donors primed with *C*. *albicans* [44]. Thus, priming macrophages via activation of the STAT1 pathway does appear to be important for memory-like innate priming with a correlation to training in context with microbial infection rather than with microbial by-products like β-glucan. The data presented herein are the first to show that *in vivo* IFN-γ priming of macrophages can extend the memory-like phenotype for weeks, providing protection against subsequent infection.

Previous studies in our lab have shown that mice with macrophages deficient in STAT1 are unable to mount a protective immune response against *C*. *neoformans* strain H99γ [16]. In the current studies, we demonstrate by whole transcriptome analysis that macrophages from protectively immunized, yet unchallenged, mice have few transcript variances between groups. These data are in line with other RNA sequencing studies that do not show transcriptional differences in trained monocytes/macrophages in their resting state [45]. Remarkably, we detected robust activation of the STAT1 pathway and interferon-related canonical pathways in protectively immunized mice as early as day one post-challenge with WT cryptococci. Previous studies indicated that at day one post-challenge with *C*. *neoformans*, pulmonary macrophages from protectively immunized mice have increased gene expression for STAT1 and downstream members of the STAT1 pathway, as well as phosphorylation of STAT1, confirming the RNA-seq analysis [19]. By day three post-challenge, pathways associated with innate/adaptive immune communication are up-regulated. Since this immune response in macrophages trained by *C*. *neoformans*/IFN-γ happens so quickly, it suggests that the innate arm of the immune system is responsible for protection. Furthermore, these studies show that protection occurs in the absence of B and T cells, as well as NK cells and neutrophils, demonstrating the critical role that macrophages play in vaccine-mediated immune responses. It must be noted that cell types not depleted, such as dendritic cells, may be playing a role in the protective immune response in the multi-cell type depleted mice. Further investigation into DC memory-like responses is ongoing.

In the *C*. *neoformans*/IFN-γ training model, macrophages and other immune cells in mice immunized with *C*. *neoformans* strain H99γ are simultaneously exposed to cryptococcal antigen and IFN-γ. IFN-γ priming of human monocytes *in vitro* can induce sustained occupancy of transcription factors STAT1, IRF-1, and associated histone acetylation at promoters and enhancers at the *TNF*, *IL6*, and *IL12B* loci [46]. In these studies, priming did not induce transcription but created a poised environment that enhanced gene transcription during secondary stimulation with LPS [46], signifying that IFN-γ/STAT1 activation is important for mediating anti-microbial responses to a variety of stimuli including TLR agonists [47]. These data are similar to what we have observed in pulmonary macrophages from protectively immunized mice in that STAT1 is bound to the promotor regions of certain IFN-γ induced genes, including *CXCL10*, *CIITA*, and *GBP6* at higher rates than in macrophages from non-protectively immunized mice. Transcription of these genes does not appear to be active as there is no difference in cytokine production in the macrophages when left unstimulated *ex vivo*.

Investigation into specific histone modifications in methylation patterns revealed that H3K27me3, H3K4me3 and, to a lesser extent, H3K9me3 are important for cytokine recall responses of macrophages following stimulation with *C*. *neoformans*. We have thus far observed that inhibition of EZH2, which tri-methylates H3K27, resulted in decreased cytokine production and inhibition of STAT1 phosphorylation. Tri-methylation of H3K4 is often associated with active transcription of genes in macrophages [48–50]. M1 macrophages up-regulate the histone methyltransferase MLL complex which adds the H3K4me3 mark [51, 52]. H3K4me3 is found at the promoter site for pro-inflammatory chemokine and M1 macrophage marker CXCL10, correlating with MLL activation and activity [52]. Furthermore, NF-κB can recruit the MLL complex to add H3K4me3 and activate transcription of the *Nos2* and *IL6* genes in *Listeria monocytogenes* infected macrophages in synergy with STAT1 [51]. In the current studies, inhibition of the MLL complex in IFN-γ trained macrophages with MI-2 resulted in decreased pro-inflammatory cytokine production, further confirming that H3K4me3 is important for promotion of M1 macrophage activity and anti-cryptococcal responses. Overall, these data emphasize that dynamic changes in histone modifications must occur upon re-stimulation with cryptococci in order to induce pro-inflammatory cytokines that are important for protection. Future studies will determine the genes targeted by specific histone modifications that occur after IFN-γ training that likely aid in the rapid response of macrophages during *in vivo* challenge with WT *C*. *neoformans*.

IFN-γ training of macrophages is a realistic novel therapeutic approach that selectively targets IFN-γ associated genes and leaves residual TLR and CLR functions intact for host-defense mechanisms [46]. Here, we demonstrate for the first time that macrophages can be primed *in vivo* with *C*. *neoformans*/IFN-γ to expediently activate the STAT1 pathway, maintain the innate memory phenotype for at least 70 days, and provide complete protection against secondary challenge. It is likely that the concurrent and sustained IFN-γ production by the genetically modified *C*. *neoformans* strain primed the macrophages to respond to *Cryptococcus* in a more specific manner compared to that observed during typical exposures. Other vaccine strategies including the use of genetically modified *Cryptococcus* strains, crude cryptococcal extracts or recombinant proteins such as mannoproteins or heat shock proteins to induce cell-mediated immune responses induced varying levels of protection against challenge [53–59]. A novel strategy using β-glucan particles as an adjuvant delivery platform packed with alkaline extracts from cryptococcal capsule mutant strains has recently shown efficacy against *C*. *neoformans* and *C*. *gattii* in a CD4^+^ T cell-dependent manner [60]. Antibody mediated immunity (AMI) contributes to protective responses; however, there is no conclusive evidence that protection can be achieved via AMI alone. Considering that the majority of individuals who succumb to cryptococcosis are T-cell deficient, innovations in the field that target host innate immune cells are potentially the best poised to protect against this fungal pathogen. Taken together, these results show that vaccines and/or immune therapies can be designed to induce innate cell memory-like immune responses that aid in protective responses among immunocompromised individuals who are most at risk of developing cryptococcosis and other life-threatening diseases.

## Materials and methods

### Ethics statement

All animal experiments were conducted following NIH guidelines for housing and care of laboratory animals and in accordance with protocols approved by the Institutional Animal Care and Use Committee (protocol number MU021) of the University of Texas at San Antonio. A scoring-system for assessment of animal distress was established before infection experiments were started. Based on these guidelines, general condition and behavior of the animals was controlled by well-educated and trained staff. Depending on the progress of the disease, animals were monitored twice daily during the “day-phase” (7:00 am to 7:00 pm). In order not to disturb the circadian rhythm of the animals, there was no monitoring after 7:00 pm. Humane endpoint by CO_2_ asphyxiation followed by cervical dislocation was conducted if death of the animals during the following hours was to be expected.

### Mice

Female BALB/c (H^-2d^) mice (National Cancer Institute/Charles River Laboratories) and female B cell KO mice C.Cg-Cd19^tm1(cre)Cgn^Igh^b^/J and control BALB/cByJ mice (The Jackson Laboratory, Bar Harbor, ME) were used throughout these studies and housed at The University of Texas at San Antonio Small Animal Laboratory Vivarium and handled according to guidelines approved by the Institutional Animal Care and Use Committee.

### Strains and media

*C*. *neoformans* strain H99, *C*. *neoformans* strain H99γ (derived from H99 serotype A, mating type α) [15], *C*. *neoformans* calcineurin mutant (*cna1*Δ, derived from H99, mating type α, and *C*. *deuterogattii* strain R265 (each kind gifts from Dr. Joseph Heitman), mCherry expressing *C*. *neoformans* strain (KN99mCH, a kind gift from Dr. Jennifer Lodge), *C*. *bacillisporus* strain WSA87, and *C*. *deneoformans* strain 52D (each kind gifts from Dr. Brian Wickes) were recovered from a 15% glycerol stock stored at -80°C prior to use in the experiments described in this study. The strains were maintained on yeast extract/peptone/dextrose (YPD) medium agar plates (Becton Dickinson, Sparks, MD). Yeast cells were grown for 16–18 h at 30°C with shaking in liquid YPD broth, collected by centrifugation, washed three times with sterile phosphate buffered saline (PBS), and viable yeasts were quantified using trypan blue dye exclusion on a hemacytometer. As a control for immunization studies, *C*. *neoformans* strain H99γ was grown, washed, and counted as stated above, then heat killed by boiling at 65^°^C for 1 hour and killing verified by plating on YPD agar (HKH99γ). For cytokine recall assays, *Staphylococcus aureus* strain UAMS-1 and *Candida albicans* SC5314 were grown in *Luria*-Bertani medium (Fisher Scientific, Houston, TX) at 37°C or YPD broth at 30°C, respectively, for 18 hours. The cultures were then washed, counted, and heat killed as described above.

### Pulmonary cryptococcal inoculations/immunizations

Mice were anesthetized with 2% isoflurane using a rodent anesthesia device (Eagle Eye Anesthesia, Jacksonville, FL) then given an intranasal inoculation with 1 X 10^4^ CFU of *C*. *neoformans* strain H99γ or heat killed *C*. *neoformans* strain H99γ in 50 μl of sterile PBS and allowed 70 days to resolve the infection. Subsequently, the immunized mice received a second experimental pulmonary inoculation with 1 × 10^4^ CFU of wild-type *C*. *neoformans* strain H99 in 50 μl of sterile PBS. The inocula used for nasal inhalation were verified by quantitative culture on YPD agar. Mice were euthanized on predetermined days by CO_2_ inhalation followed by cervical dislocation, and lung tissues were excised using aseptic technique. Alternatively, mice intended for survival analysis were monitored by inspection twice daily and euthanized if they appeared to be in pain or moribund.

### Cell depletions

Mice were depleted of CD4^+^ and/or CD8^+^ T cell subsets via intraperitoneal (ip) administration of 200 μg anti-CD4 (GK1.5, rat IgG2b) and 200 μg anti-CD8α (2.43, rat IgG2b) antibodies (National Cell Culture Center) in 200 μl PBS as previously described beginning at 48 hours prior to challenge and administered weekly [11]. For neutrophil depletions each mouse received ip injections of 200 ug anti-Ly6G (1A8)(BioXcell) in 100 μl every other day. For NK cell depletion, each mouse received ip injections of 0.5 ug anti-asialo-GM (Wako USA) every 3 days. Neutrophil and NK cell depletions were started 24 hours prior to challenge. Control IgG2a and IgG2b isotype control antibodies were used (eBioscience Inc., San Diego, CA). All depletions were maintained through the entirety of the study at concentrations and schedules chosen following studies testing different dosages and schedules to determine the optimum dosage and schedule for each antibody. The efficiency of all cell depletions in the lungs and spleens was assessed by flow cytometric analysis using antibodies that adhere to epitopes distinct from those adhered to by the depletion antibodies (S1 Fig). Antibodies depleted approximately 95% of neutrophils, 87% of NK cells, 98% of CD4^+^ T cells, and 98% of CD8^+^ T cells.

### Flow cytometry

Lungs were excised and digested enzymatically at 37°C for 30 min in 10 ml digestion buffer (RPMI 1640 and 1 mg/ml collagenase type IV (Sigma-Aldrich, St. Louis, MO) with intermittent (every 10 min) stomacher homogenizations. The digested tissues were then successively filtered through sterile 70- and 40-μm nylon filters (BD Biosciences, San Diego, CA) to enrich for leukocytes, and then cells were washed with sterile HBSS. Erythrocytes were lysed by incubation in NH_4_Cl buffer (0.859% NH_4_Cl, 0.1% KHCO_3_, 0.0372% Na_2_EDTA [pH 7.4]; Sigma-Aldrich) for 3 min on ice followed by a 2-fold excess of PBS. Standard methodology was employed for the direct immunofluorescence of pulmonary leukocytes as previously described [11]. Cells were incubated with CD16/CD32 (Fc Block; BD Biosciences) and fluorophore-conjugated antibodies were used to stain for cell surface staining (S3 Table). The samples were acquired on a BD FACSArray flow cytometer (BD Biosciences) and data analyzed using FlowJo Software (Ashland, OR).

### Macrophage enrichment

A single cell suspension of pulmonary leukocytes was acquired as described above. The resulting leukocyte population was then depleted of CD3^+^ cells using an α-CD3 microbead kit (Miltenyi Biotec, Auburn, CA), then enriched for macrophages using biotinylated α-F4/80 and subsequent binding of anti-biotin magnetic beads (Miltenyi Biotec) according to the manufacturer’s recommendations. Splenic macrophages were isolated as described for the pulmonary macrophages, omitting the collagenase digestion. Purity was verified using Labeling Check Reagent-APC (Miltenyi Biotec) and F4/80-PE on a BD FACSArray Flow Cytometer (BD Biosciences) as described above and purity of 85–95% was routinely achieved with less than 3% T cells detected.

### Reagents/Chemicals

Reagents used were as follows: LPS (*Escherichia coli* O111:B4, Sigma Aldrich); GSK 343, MI-2 (hydrochloride), UNC0638 and Torin 1 (Cayman Chemical, Ann Arbor, MI); MTA (5’-Deoxy-5’-(methylthio)adenosine) and pargyline (Sigma Aldrich); EGCG ((-)-Epigallocatechin gallate) and givinostat (ITF2357) (Selleck Chemicals, Houston, TX).

### Cytokine recall assays

Splenic macrophages (5 × 10^5^/well) derived from mice immunized with *C*. *neoformans* strain H99γ or HKH99γ on day 70 post-immunization or naive female BALB/c mice were cultured in RPMI complete media with or without *C*. *neoformans cna1*Δ, heat killed *C*. *neoformans* H99, heat killed *C*. *deuterogattii* R265, *C*. *bacillisporus* WSA87, or heat killed *C*. *deneoformans* 52D (at a 1:1 ratio) at 37°C and 5% CO2 in 96 well round-bottom culture plates (Becton Dickinson Labware, Franklin Lakes, NJ). The culture supernatants were collected after 24 h, protease and phosphatase inhibitor cocktail (Thermo Fisher, Rockford, IL) was added and samples were stored at -20°C until analysis. Cytokine production was analyzed using the Bio-Plex protein array system (Luminex-based technology; Bio-Rad Laboratories, Hercules, CA). Alternatively, splenic macrophages were cultured in media alone, media +/- dimethyl sulfoxide (DMSO; Fisher Scientific), dimethylformamide (DMFM; Fisher Scientific), and various histone modification inhibitors or in media containing *C*. *neoformans cna1*Δ, 0.3 ug/ml LPS, heat killed *S*. *aureus* (5 × 10^5^/well) or heat killed *C*. *albicans* (5 × 10^5^/well) +/- histone modification inhibitors for 24 h and cytokine levels in supernatants were analyzed by Bio-Plex Pro^tm^ Mouse Cytokine Th1/Th2 8-plex Assay (Bio-Rad) or IL-2 ELISA (Affymetrix). Culture of macrophages with the carriers DMSO and DMFM resulted in cytokine production similar to media alone.

#### Phagocytosis/Association assay

Pulmonary macrophages from either HKH99γ or H99γ immunized mice were isolated as described above. Macrophages were plated at a concentration of 1 x 10^6^ cells and incubated with an mCherry expressing strain of *Cryptococcus*, KN99mCH, at a 5:1 ratio (*Cn* to macrophage) for 6 h. Macrophages were incubated in 1X PBS and Zombie viability dye (BioLegend) for 20 minutes. Cells were then washed and macrophages were labeled with CD11b and CD64 for 30 mins at 4^°^C, washed with FACS buffer and then fixed in 2% ultra-pure formaldehyde (Life Technologies). Analysis was performed in triplicate and analyzed using ImageStreamX IDEAS 6.2 software (Millipore) after 100,000 cells were collected.

#### Intracellular cytokine staining

Pulmonary macrophages were isolated as described above. Macrophages were then incubated with *C*. *neoformans cna1*Δ at a 1:1 ratio at 37°C in 5% CO_2_ in RPMI without phenol red plus 10% heat-inactivated fetal bovine serum (Life Technologies) for 2 h. Golgi plug (Brefeldin A; BD Biosciences) was added according to manufacturer's recommendations and incubated for an additional 4 h. (6 h. total). Cells were washed with 1X PBS and stained with Zombie viability dye (Cat No 423104; Biolegend) at room temperature in the dark for 15 mins then washed with 1x PBS. Cells were stained for surface markers CD45, CD64, and CD11b (S3 Table) and incubated at 4°C for 30 mins. Cells were washed and fixed with Fix/Permeabilization buffer (BD Biosciences) for 20 mins. Cells were washed with 1X Perm/wash buffer (BD Biosciences) and stained for intracellular markers IFN-γ, IL-2, and TNF-α (S3 Table) for 30 min at 4°C. Cells were washed with 1X perm/wash buffer and fixed with 2% ultra-pure formaldehyde (Life Technologies). Analysis was performed in triplicate and analyzed using ImageStreamX IDEAS 6.2 software (Millipore) after 100,000 cells were collected.

### Western blot

For Western blotting of macrophages were stimulated as described above. After stimulation and collection of supernatants, cells were lysed in 35 μl of lysis buffer (Millipore) and biological replicates pooled. Equal amounts of protein were subjected to SDS-PAGE electrophoresis. Primary antibodies [1:500 and 1:50 000 (β-actin)] in 5% (w/v) BSA/TBST (5% bovine serum albumin/TBST) were incubated overnight at 4°C. HRP-conjugated anti-rabbit antibody at a dilution of 1:5000 in 5% (w/v) BSA/TBST was used for 1 hour at room temperature. The following antibodies were used: β-actin antibody, mTOR antibody, phospho-mTOR antibody (Ser2448), STAT1, and phosphor-STAT1 (Tyr701) antibody (Cell Signaling, Leiden, Netherlands; S3 Table). At least 3 different individual experiments were repeated for each Western blot experiment.

### RNA purification and sequencing

Total RNA was isolated from purified pulmonary F4/80^+^ cells using TRIzol reagent (Invitrogen, Carlsbad, CA) and then DNase (Qiagen, Germantown, MD) treated to remove possible traces of contaminating DNA according to the manufacturer’s instructions. Total RNA was subsequently recovered using the Qiagen RNeasy kit. RNA integrity and concentration was assessed via Bioanalyzer using the Agilent RNA 6000 Nano Kit according to the manufacturer’s recommendations (Agilent, Santa Clara, CA). Minimum acceptable RNA integrity number (RIN) was set at 7 for use in RNA-sequencing studies. Library preparation and sequencing were performed by UT Southwestern Medical Center Genomics and Microarray Core Facility, Dallas, TX. Resulting RNA sequences were deposited in the Sequence Read Archive, PRJNA420072.

### Gene expression analysis

Data normalization and differential gene expression was determined using a method that incorporates an internal-standard based approach of normalization and an associative t-test to minimize false positive determinations as previously described [61, 62] and performed by UT Southwestern Medical Center Genomics and Microarray Core Facility, Dallas TX. Genes exhibiting normalized expression values 20 times the standard deviation of the statistically defined background were considered expressed. Genes differentially expressed ≥2 fold passed the standard t-test significance level of p<0.05 and passed an associative t-test threshold to eliminate false positive determinations.

### Pathway and network analysis

Functional pathway and network analyses of differentially expressed genes were performed using Ingenuity Pathway Analysis (IPA) (Qiagen, Redwood City, CA). The Ingenuity Knowledge Base, a repository of biological interactions, was used as a reference set. The functional analysis module in IPA was used to identify over-represented molecular and cellular functions of differentially expressed genes. The probability that each biological function assigned to the data set was due to chance alone was estimated, and a false discovery rate (FDR) <0.05 was used to correct for multiple comparisons. Over-represented canonical signaling and metabolic pathways in the input data were determined based on two parameters: (1) The ratio of the number of molecules from the focus gene set that map to a given pathway divided by the total number of molecules that map to the canonical pathway, and (2) a P-value calculated by Fisher’s exact test that determines the probability that the association between the focus loci and the canonical pathway is explained by chance alone. Network analysis used focus genes as “seeds” to infer *de novo* interaction networks. Direct interactions between focus loci and other molecules were inferred based on experimentally observed relationships supported by at least one reference from the literature. Additional molecules from the Ingenuity Knowledge Base were added to the network to fill or join smaller networks. The network score was based on the hypergeometric distribution and calculated with the right-tailed Fisher’s exact test. A higher score indicates a lower probability of finding the observed number of focus molecules in a given network by chance.

### Gene ontology analysis

Gene ontology analysis was performed using the DAVID functional analysis tool [63]. The Bonferroni, Benjamini, and FDR (false discovery rate) were used for multiple test correction.

### Chromatin immunoprecipitation

Pulmonary F4/80^+^ cells (5 x 10^6^) isolated from immunized mice were crosslinked, lysed, and sonicated with the Bioruptor ultrasonicator (Diagenode, Denville, NJ). 10% of chromatin was reserved as input control. The remaining chromatin was immunoprecipitated with antibodies against Normal IgG or STAT1 (Cell Signaling Technology, Danvers, MA) bound to Protein A/G Magnetic Beads (Pierce Biotechnology, Rockford, IL). Immunoprecipitated DNA and Input DNA was reverse crosslinked, eluted, and purified with the IPure kit v2 (Diagenode) according to manufacturer’s instructions.

### Real time ChIP qPCR arrays

Immunoprecipitated DNA was used as a template for real time ChIP qPCR analysis using EpiTect ChIP Custom qPCR Arrays (Qiagen). Genes were chosen based on RNA-seq day one post-challenge IPA analysis. Primer sequences for regions 1kb upstream of the transcription start site of IRF-1, iNOS, GBP2, GBP5, GBP6, CXCL9, CXCL10, CXCL11, SOCS-1, CIITA, IFIT2, and IFI47 were used in the array. A master mix consisting of 2ul DNA per 25ul reaction was mixed with RT^2^ SYBR Green qPCR master mix (Qiagen) according to manufacturer’s recommendations and Real Time ChIP qPCR arrays were performed using 7300 real-time PCR System (Applied Biosystems, Foster City, CA). Percent IP was calculated based on comparative threshold values using the SuperArray ChIP-qPCR Data Analysis Template supplied by Qiagen according to manufacturer’s instructions.

### Statistical analysis

Survival data was analyzed using the log-rank test (GraphPad Software). The unpaired Student’s t test was used to analyze comparisons between two groups to detect statistically significant differences. For multiple comparisons, a one-way ANOVA or two-way ANOVA with the Tukey’s multiple comparison test was performed. Significant differences were defined as *p < 0.05, **p < 0.01 or ***p < 0.001.

## Supporting information

S1 Fig Flow cytometry analysis of cell depletions. B cell knock out mice were immunized with *C*. *neoformans* strain H99γ and rested for 70 days. Mice were subsequently treated with isotype control antibodies or depleted of CD4^+^ T cells, CD8^+^ T cells, NK cells, and/or neutrophils prior to challenge with wild type *C*. *neoformans*. Cell depletions were maintained throughout the observation period. Depletion of the various cell types in the lungs and spleen was confirmed by flow cytometry upon termination of the survival study. Data shown is from pooled lungs of 5–7 mice per group.
(TIF)

S2 Fig Flow cytometry analysis of intracellular cytokine production. BALB/c mice were immunized with *C*. *neoformans* strain H99γ and rested for 70 days. Macrophages were isolated from the lungs and spleens and cultured *ex vivo* with *C*. *neoformans cna1*Δ. Production of cytokines TNF-α, IFN-γ and IL-2 were verified by intracellular flow cytometry after 6 hours of culture, left panel and histograms. The right panel establishes that the cytokine production is not detected in unstimulated macrophages from naïve mice, demonstrating that the cytokine production observed in the left panel is in response to stimulation with *cna1*Δ. Data shown is representative of 3 individual experiments with 10 mice per group.
(PDF)

S1 Table Gene ontology of RNA-seq in macrophages from H99γ immunized compared to HKH99γ immunized mice 1 day post-challenge. Pulmonary macrophages from H99γ and HKH99γ immunized mice were isolated at day 1 post-challenge with C. neoformans strain H99. RNA was extracted from the macrophages, sequenced, and gene ontology analysis was performed using DAVID functional analysis tool.
(PDF)

S2 Table Gene ontology of RNA-seq in macrophages from H99γ immunized compared to HKH99γ immunized mice 3 days post-challenge. Pulmonary macrophages from H99γ and HKH99γ immunized mice were isolated at day 3 post-challenge with C. neoformans strain H99. RNA was extracted from the macrophages, sequenced, and gene ontology analysis was performed using DAVID functional analysis tool.
(PDF)

S3 Table Antibodies. Antibodies used for flow cytometric analysis and western blot analysis.
(PDF)
