# Supplementary figures and images for: IFN-γ immune priming of macrophages in vivo induces prolonged STAT1 binding and protection against Cryptococcus neoformans
Source: PLoS Pathog. 2018 Oct 10;14(10):e1007358. doi: 10.1371/journal.ppat.1007358 (PMC6197699; doi:10.1371/journal.ppat.1007358)

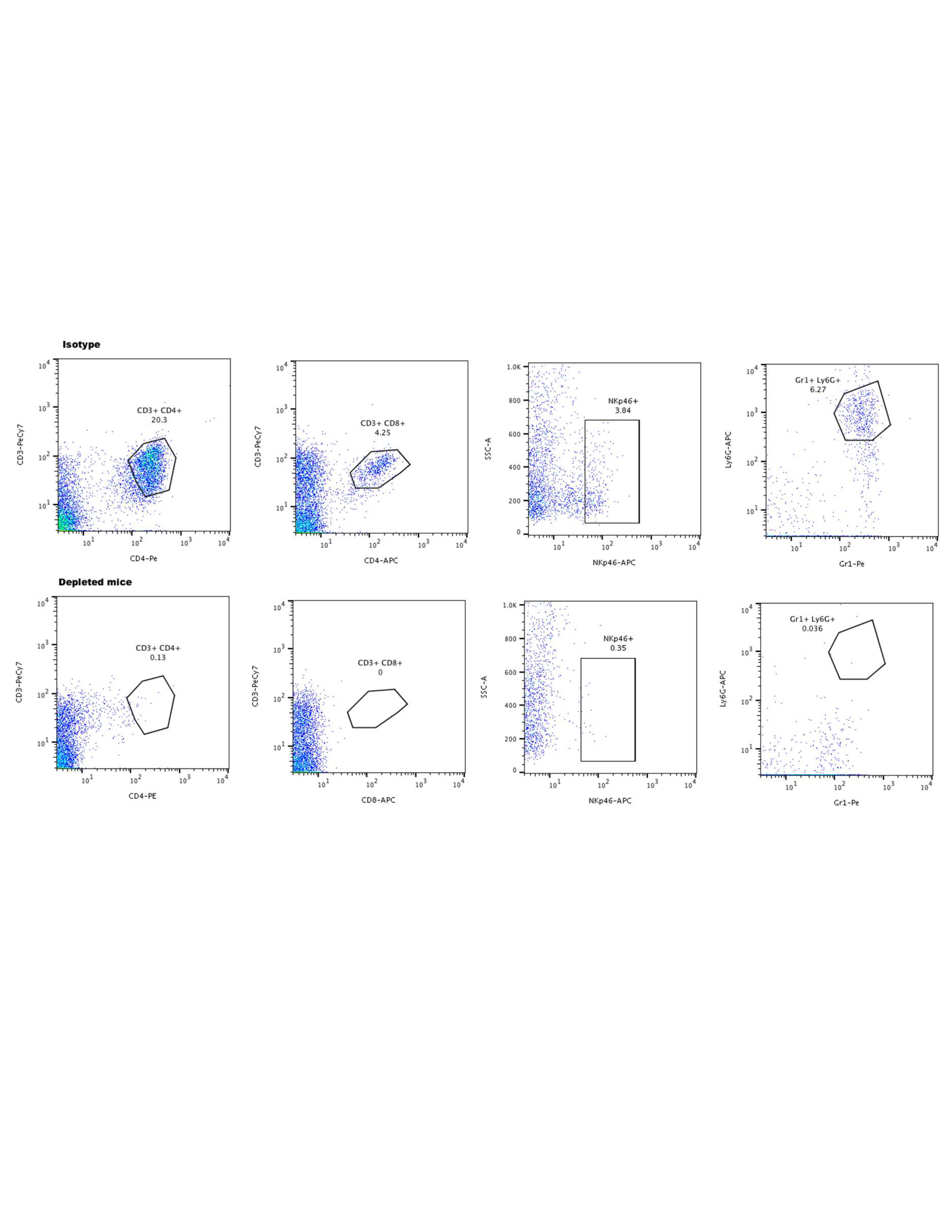

Supplement: S1 Fig — B cell knock out mice were immunized with C. neoformans strain H99γ and rested for 70 days. Mice were subsequently treated with isotype control antibodies or depleted of CD4+ T cells, CD8+ T cells, NK cells, and/or neutrophils prior to challenge with wild type C. neoformans. Cell depletions were maintained throughout the observation period. Depletion of the various cell types in the lungs and spleen was confirmed by flow cytometry upon termination of the survival study. Data shown is from pooled lungs of 5–7 mice per group. (TIF) [file ppat.1007358.s001.tif]
