# Supplementary material for: IFN-γ immune priming of macrophages in vivo induces prolonged STAT1 binding and protection against Cryptococcus neoformans
Source: PLoS Pathog. 2018 Oct 10;14(10):e1007358. doi: 10.1371/journal.ppat.1007358 (PMC6197699; doi:10.1371/journal.ppat.1007358)
Supplement: S2 Fig — BALB/c mice were immunized with C. neoformans strain H99γ and rested for 70 days. Macrophages were isolated from the lungs and spleens and cultured ex vivo with C. neoformans cna1Δ. Production of cytokines TNF-α, IFN-γ and IL-2 were verified by intracellular flow cytometry after 6 hours of culture, left panel and histograms. The right panel establishes that the cytokine production is not detected in unstimulated macrophages from naïve mice, demonstrating that the cytokine production observed in the left panel is in response to stimulation with cna1Δ. Data shown is representative of 3 individual experiments with 10 mice per group. (PDF) [file ppat.1007358.s002.pdf]

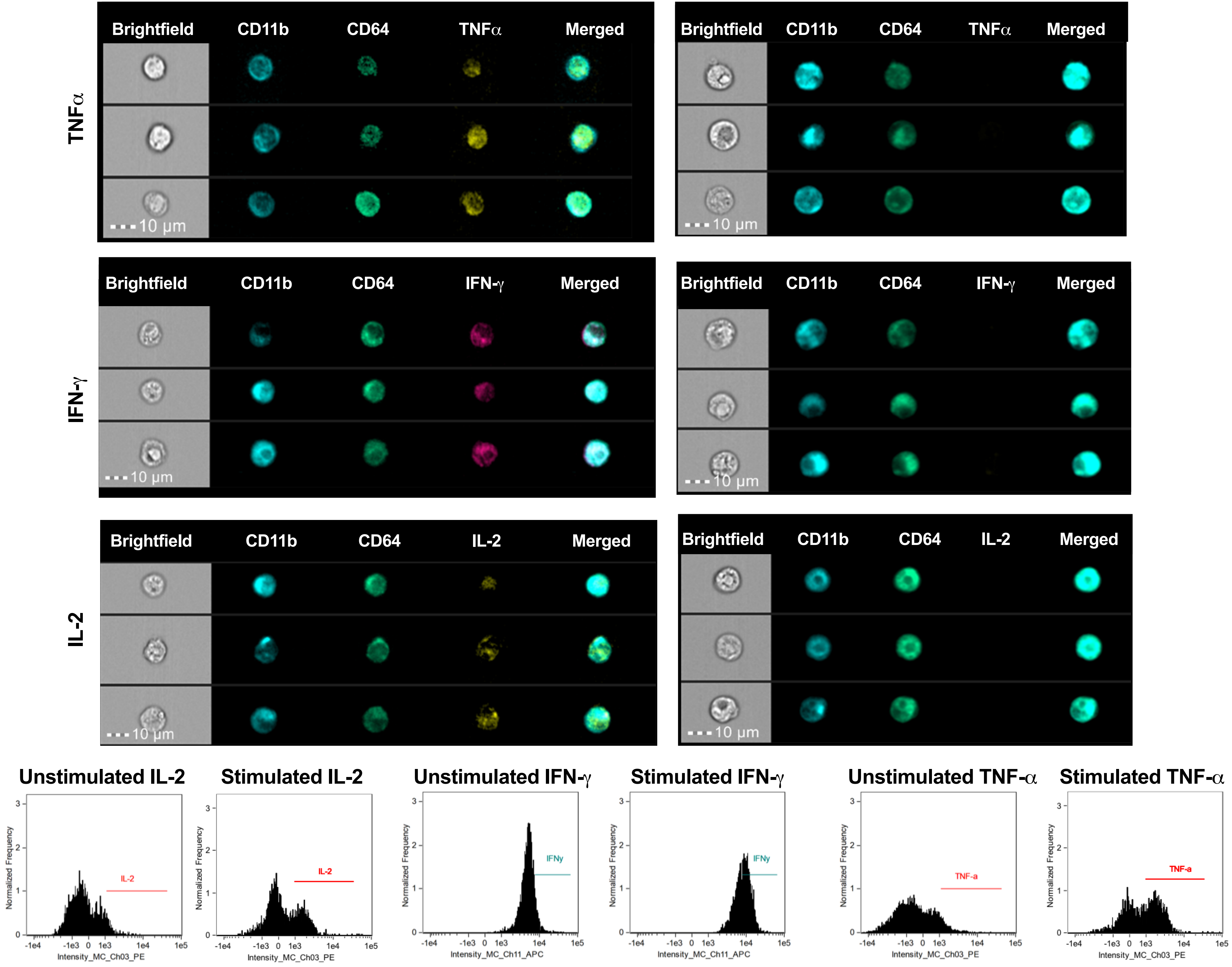
TNFα
Brightfield
CD11b
CD64
TNFα
Merged
10 µm
IFN-γ
IFN-γ
IL-2
IL-2
Unstimulated IL-2
Stimulated IL-2
Unstimulated IFN-γ
Stimulated IFN-γ
Unstimulated TNF-α
Stimulated TNF-α
Normalized Frequency
Intensity_MC_Ch03_PE
Intensity_MC_Ch11_APC
IFNγ
TNF-a
