# Supplementary material for: IFN-γ immune priming of macrophages in vivo induces prolonged STAT1 binding and protection against Cryptococcus neoformans
Source: PLoS Pathog. 2018 Oct 10;14(10):e1007358. doi: 10.1371/journal.ppat.1007358 (PMC6197699; doi:10.1371/journal.ppat.1007358)
Supplement: S1 Table — Pulmonary macrophages from H99γ and HKH99γ immunized mice were isolated at day 1 post-challenge with C. neoformans strain H99. RNA was extracted from the macrophages, sequenced, and gene ontology analysis was performed using DAVID functional analysis tool. (PDF) [file ppat.1007358.s003.pdf]

Supplementary Table 1: Gene ontology of RNA-seq in macrophages from H99γ immunized compared to HKH99γ immunized mice 1 day post-challenge

Term	Count	%	PValue	Genes	Fold Enrichment	Bonferroni	Benjamini	FDR
GO:0006955~immune response	31	29.81	1.11E-24	IGHG1, IL1F9, CXCL9, RSAD2, TNFSF14, CCL8, OAS2, CXCL11, CXCL10, OASL2, IFNG, TAP1, OASL1, GBP10, BCL3, LTA, GBP8, IRGM1, IL6, GBP6, GBP5, IL27, GBP9, TNFRSF14, CCL12, IL18BP, CCR5, SERPINA3G, GBP4, XCL1, GBP3, GBP2	11.76746005	1.03E-21	1.03E-21	1.74E-21
GO:0006952~defense response	18	17.31	2.43E-10	IGHG1, IL6, IRGM1, IL27, CXCL9, CCL8, RSAD2, IFI47, CXCL11, CXCL10, CCL12, MEFV, CCR5, IFNG, TAP1, BCL3, NOS2, LTA	7.183505639	2.24E-07	1.12E-07	3.79E-07
GO:0005125~cytokine activity	12	11.54	6.38E-09	IL1F9, CCL12, IL6, IL27, IFNG, CXCL9, TNFSF14, CCL8, XCL1, CXCL11, LTA, CXCL10	11.35726496	1.07E-06	1.07E-06	7.76E-06
GO:0005615~extracellular space	16	15.38	2.41E-08	IGHG1, IL1F9, IL6, IL27, CXCL9, CCL8, TNFSF14, CXCL11, CXCL10, CCL12, EREG, IFNG, IL15RA, HBEGF, XCL1, LTA	6.11741683	1.81E-06	1.81E-06	2.53E-05
GO:0006954~inflammatory response	12	11.54	3.88E-08	IGHG1, CCL12, IL6, CCR5, MEFV, IL27, CXCL9, CCL8, NOS2, CXCL11, LTA, CXCL10	9.535438596	3.58E-05	1.19E-05	6.07E-05
GO:0009611~response to wounding	14	13.46	4.77E-08	IGHG1, CCL12, IL6, MEFV, CCR5, IL27, F3, CXCL9, HBEGF, CCL8, NOS2, CXCL11, LTA, CXCL10	7.21340816	4.40E-05	1.10E-05	7.45E-05
GO:0003924~GTPase activity	10	9.615	5.00E-08	GBP8, GBP6, IGTP, GBP5, IRGM2, GBP9, GBP10, IIGP1, GBP4, GBP3, GBP2	13.30929487	8.34E-06	4.17E-06	6.08E-05
GO:0005525~GTP binding	14	13.46	1.08E-07	GBP8, GBP6, IRGM1, IRGM2, GBP5, GBP9, IFI47, GM12250, IGTP, GBP10, IIGP1, GBP4, GM4951, GBP3, GBP2	6.737360568	1.80E-05	6.00E-06	1.31E-04
GO:0032561~guanyl ribonucleotide binding	14	13.46	1.44E-07	GBP8, GBP6, IRGM1, IRGM2, GBP5, GBP9, IFI47, GM12250, IGTP, GBP10, IIGP1, GBP4, GM4951, GBP3, GBP2	6.57031857	2.41E-05	6.02E-06	1.75E-04
GO:0005576~extracellular region	26	25	1.92E-07	IGHG1, IL1F9, CXCL9, TNFSF14, CCL8, CXCL11, MMP25, TIMP1, CXCL10, ISG15, IFNG, SEMA3E, IL15RA, FGL2, LTA, IL6, VSTM2A, IL27, MMP14, CCL12, WFDC10, IL18BP, EREG, HBEGF, XCL1, LIPF	3.023660714	1.44E-05	4.80E-06	2.02E-04
GO:0008009~chemokine activity	6	5.769	2.48E-06	CCL12, CXCL9, CCL8, XCL1, CXCL11, CXCL10	26.89878543	4.13E-04	8.27E-05	0.00301
GO:0019221~cytokine-mediated signaling pathway	6	5.769	1.37E-05	IL2RB, IL6, EREG, SOCS1, IIGP1, STAT1	19.15601504	0.012591304	0.002531	0.02147
GO:0009986~cell surface	10	9.615	2.02E-05	IL2RB, IL6, CCR5, KLRK1, EMR4, TNFRSF14, MMP14, CD209B, HTR2C, MMP25	6.405737705	0.001515074	3.79E-04	0.02121

GO:0009617~response to bacterium	8	7.692	2.47E-05	IGHG1, CCR5, IFNG, BCL3, NOS2, STAT1, CD209B, IRG1	9.110291653	0.022547461	0.0037937	0.03863
GO:0042035~regulation of cytokine biosynthetic process	6	5.769	2.65E-05	IL6, EREG, IFNG, IRF1, BCL3, CD209B	16.76151316	0.024143196	0.0034853	0.0414
