# Supplementary material for: IFN-γ immune priming of macrophages in vivo induces prolonged STAT1 binding and protection against Cryptococcus neoformans
Source: PLoS Pathog. 2018 Oct 10;14(10):e1007358. doi: 10.1371/journal.ppat.1007358 (PMC6197699; doi:10.1371/journal.ppat.1007358)
Supplement: S2 Table — Pulmonary macrophages from H99γ and HKH99γ immunized mice were isolated at day 3 post-challenge with C. neoformans strain H99. RNA was extracted from the macrophages, sequenced, and gene ontology analysis was performed using DAVID functional analysis tool. (PDF) [file ppat.1007358.s004.pdf]

Supplementary Table 2: Gene ontology of RNA-seq in macrophages from H99γ immunized compared to HKH99γ immunized mice 3 days post-challenge

Term	Count	%	PValue	Genes	Fold Enrichment	Bonferroni	Benjamini	FDR
GO:0006955~immune response	106	12.8484848	2.1697E-48	IL18, TLR1, TNFSF14, CXCL11, C1QC, TLR9, CXCL10, B2M, C1RA, PGLYRP2, OASL2, IFNG, OASL1, GBP10, MX1, MX2, GBP8, GBP6, GBP5, C4B, IL27, GBP9, H2-DMB1, TNFRSF14, C1QB, CCR5, GM10499, CCR2, OAS1B, OAS1A, GBP4, GBP3, OAS1G, GBP2, IFIH1, MILL2, OAS3, CCL8, SP110, OAS2, CX3CL1, CCL5, CD74, ADA, CCL6, SLC11A1, GM8810, PROCR, H2-T10, DHX58, TLR12, H2-Q5, CD8B1, CFB, H2-Q1, TINAGL1, H2-Q6, H2-Q7, FCGR1, PSMB8, PSMB9, H2-EB2, CXCL13, H2-EB1, H2-T22, H2-T23, H2-T24, IGHG1, CD8A, CD1D1, SKAP1, CD1D2, NOD2, TMEM173, SH2D1A, LTA, H2-K1, IL18RAP, SLA2, IL18BP, LAX1, SERPINA3G, H2-BL, IL12A, H2-AA, IL1F9, CSF2, CXCL5, C3, CXCL9, RSAD2, PF4, C1S, MIF, CCL24, TAP2, TAP1, BCL3, CD4, IRGM1, H2-M3, OLR1, SAMHD1, H2-AB1, CCL17, CCL12, C920025E04RIK, FCGR2B, IRF7, IRF8, C1RL, RNF19B	5.39333551	5.6478E-45	5.6478E-45	3.8452E-45
GO:0006952~defense response	76	9.21212121	7.0951E-26	IGHG1, TLR1, PPARG, CD1D1, CXCL11, C1QC, TLR9, CD1D2, B2M, CXCL10, C1RA, NOD2, TMEM173, SH2D1A, PGLYRP2, IFNG, NOS2, DDAH2, MX1, MX2, LTA, H2-K1, IL18RAP, C4B, IL27, SAA3, IFI47, C1QB, SERPINA3N, HIF1A, CCR5, CCR2, PLA2G7, H2-AA, PTAFR, IFIH1, CXCL5, C3, CXCL9, CCL8, RSAD2, SP110, C1S, CCL5, CD24A, CD74, TRF, MIF, CCL24, SLC11A1, IL17A, MEFV, TAP1, IL17F, PYCARD, BCL3, DHX58, TLR12, BMP2, IRGM1, LIPA, OLR1, H2-M3, CFB, H2-Q1, SAMHD1, H2-Q6, H2-Q7, FCGR1, CCL17, ORM1, CCL12, AY761185, FCGR2B, CXCL13, IRF8, C1RL, ALOX5, BMP6	4.0654447	1.8469E-22	9.2343E-23	1.2574E-22
GO:0005886~plasma membrane	233	28.2424242	5.8529E-24	ADCY3, EFNA1, AIF1, SLC9A4, FGFRL1, L1CAM, CD52, CD209B, AQP1, B2M, LPHN2, LPHN3, PRRT1, ADAM8, GBP5, EFNB2, CD40, SSPN, PDCD1LG2, THY1, JUP, KRT19, RND1, PGM5, CD36, PTRF, CCR5, HTR7, CCR2, HS3ST3B1, MILL2, NRN1, EPHB4, CD74, GPR141, RIMS3, GM8810, ACE, CD69, SLC28A2, CDC42EP5, CD8B1, MAOA, UPK3B, MCAM, SECTM1A, FCGR1, ABCG1, CAMK2N1, PKP3, NOTCH4, SYTL3, TREML2, IGHG1, GPR84, STEAP4, GYPC, LIMA1, ART2B, SLC15A2, GJA4, KCNIP4, MMP25, ART3, NOD2, GPIHBP1, ATP6V0D2, CEACAM1, LAG3, GPR97, HYAL2, CMKLR1, GPR171, MMP15, MMP14, LAX1, H2-BL, KCTD12B, SGCE, GPR182, NKD1, FFAR2, CSF1, GPR65, FPR1, KIT, LY6A, SORBS3, LY6E, MFSD7C, SLCO3A1, AMICA1, RASA4, KCNE3, IRGM1, SELL, H2-AB1, LY6I, FCGR2B, C920025E04RIK, SLC16A9, HBEGF, PERP, TAPBPL, TJP2, HTR2C, LRP4, SLC22A17, OCLN, SLC22A18, SLC6A4, TLR1, CSPG4, TNFSF14, TLR9, DYSF, A630033H20RIK, MCOLN3, SLC2A1, ROBO4, IL15RA, ELTD1, INADL, MYO6, BST2, STAP2, H2-DMB1, TNFRSF14, TNS3, TNS1, THBD, CLEC12A, GM10499, DLL4, CAR4, GBP2, CAV2, CAV1, ADORA2B, CYSLTR2, ADORA2A, KLRK1, CX3CL1, SRC, SLCO2A1, IL12RB1, TTYH2, TEK, H2-T10, LPL, H2-Q5, ACY3, PODXL, H2-Q1, SLCO2B1, H2-Q6, H2-Q7, SHANK3, TIGIT, GPR34, H2-EB2, LTB4R1, CD274, ECSCR, H2-EB1, PRICKLE2, H2-T22, H2-T23, H2-T24, CACNA1D, PRX, IER3, CD8A, LEPR, CLDN5, CXCR1, BCAM, CXCR3, SYNGR1, CD1D1, TENC1, SKAP1, CD1D2, EPCAM, PCDH1, TMEM173, RHOC, CALCRL, TIE1, SCN7A, NRG1, GPC1, H2-K1, SLA2, SLC9A3R2, TRPM2, TMEM67, ADRB1, LCK, CLDN1, H2-AA, PTAFR, CPM, RSAD2, PLVAP, CD24A, APLP2, CDH5, APLP1, PRSS8, VCAM1, APLNR, CD9, MARVELD2, RASGRP1, TGM1, TNFRSF18, TGM2, ENO2, CD2, CD4, IL2RG, CD5, ENO1, LY6C1, MS4A4B, ICA1, IL2RB, IL2RA, SLC12A2, H2-M3, OLR1, LY6C2, VWF, KCNN4, GPR114, JAK2, EPOR, GPR116	1.81294298	1.8612E-21	1.8612E-21	7.8908E-21

GO:0009611~response to wounding	63	7.63636364	5.0568E-23	IGHG1, TLR1, PPARG, CXCL11, C1QC, TLR9, CXCL10, C1RA, NOD2, DYSF, NOS2, LTA, F10, GATM, C4B, IL27, SAA3, C1QB, SERPINA3N, HIF1A, THBD, CCR5, F3, CCR2, PLA2G7, PTAFR, CXCL5, C3, CXCL9, CCL8, NINJ1, PF4, CX3CL1, C1S, CCL5, CD24A, TRF, MIF, CCL24, ARG1, SLC11A1, IL17A, MEFV, PROCR, IL17F, PYCARD, PAPSS2, TLR12, BMP2, LIPA, OLR1, CFB, FCGR1, SOD2, ORM1, CCL12, VWF, CXCL13, C1RL, HBEGF, ID3, ALOX5, BMP6	4.3509446	1.3163E-19	4.3876E-20	8.9617E-20
GO:0006954~inflammatory response	48	5.81818182	1.4342E-20	IGHG1, CXCL5, C3, PPARG, TLR1, CXCL9, CCL8, C1S, CCL5, CXCL11, C1QC, CD24A, TRF, TLR9, MIF, CXCL10, CCL24, C1RA, SLC11A1, IL17A, NOD2, MEFV, IL17F, PYCARD, NOS2, LTA, TLR12, BMP2, LIPA, OLR1, C4B, CFB, IL27, SAA3, FCGR1, C1QB, CCL12, ORM1, SERPINA3N, HIF1A, CCR5, CXCL13, CCR2, PLA2G7, C1RL, ALOX5, PTAFR, BMP6	5.11247501	3.7333E-17	9.3332E-18	2.5417E-17
GO:0009986~cell surface	57	6.90909091	2.5124E-20	CD8A, CSPG4, L1CAM, CD1D1, SDC4, CD209B, MMP25, CD1D2, NOD2, ROBO4, GPIHBP1, GPC1, LAG3, CEACAM1, H2-K1, TNFRSF14, CD40, MMP15, MMP14, THY1, CD38, CD36, CCR5, CLEC12A, H2-BL, H2-AA, KLRK1, CX3CL1, KIT, CD24A, CD74, LY6A, SLC11A1, GM8810, IL12RB1, CD69, TNFRSF18, CD2, CD4, IL2RG, SLCO3A1, CD5, LY6C1, IL2RB, IL2RA, H2-Q5, CD8B1, H2-M3, SELL, H2-Q1, H2-AB1, H2-Q6, FCGR1, H2-Q7, ABCG1, LY6C2, TIGIT, VWF, FCGR2B, NOTCH4, CD274, HTR2C	4.22570183	7.9893E-18	3.9947E-18	3.3871E-17
GO:0019882~antigen processing and presentation	30	3.63636364	4.4904E-19	MILL2, CD1D1, CD74, CD1D2, B2M, GM8810, SLC11A1, PROCR, TAP2, IFNG, H2-T10, H2-K1, H2-Q5, H2-M3, H2-Q1, H2-DMB1, H2-AB1, H2-Q6, H2-Q7, FCGR1, PSMB8, PSMB9, H2-EB2, FCGR2B, PSME1, C920025E04RIK, PSME2, GM10499, H2-BL, H2-EB1, H2-AA, H2-T22, H2-T23, H2-T24, TAPBPL	8.26369884	1.1688E-15	2.3377E-16	7.9579E-16
GO:0002684~positive regulation of immune system process	42	5.09090909	2.6735E-17	IGHG1, ADORA2B, C3, IL18, KLRK1, TNFSF14, C1S, CD1D1, CD24A, C1QC, PNP, CD74, ADA, CD1D2, B2M, C1RA, SLC11A1, NOD2, SH2D1A, TAP2, IFNG, CD4, IL2RG, CD5, LAG3, H2-K1, IL2RA, H2-M3, C4B, CFB, EFNB1, H2-Q1, CD40, H2-Q6, FCGR1, H2-Q7, PDCD1LG2, THY1, C1QB, CD38, LAX1, LCK, C1RL, IL12A, H2-AA	4.88601223	6.9592E-14	1.1599E-14	4.738E-14
GO:0009897~external side of plasma membrane	43	5.21212121	3.2193E-17	CD8A, KLRK1, CSPG4, L1CAM, KIT, CD1D1, CD209B, CD24A, CD74, CD1D2, LY6A, IL12RB1, CD69, ROBO4, TNFRSF18, CD2, CD4, IL2RG, CD5, GPC1, LAG3, H2-K1, LY6C1, IL2RB, H2-Q5, IL2RA, CD8B1, H2-M3, SELL, H2-Q1, H2-AB1, TNFRSF14, CD40, MMP15, MMP14, H2-Q6, FCGR1, H2-Q7, ABCG1, LY6C2, THY1, VWF, CCR5, FCGR2B, CD274, H2-AA, HTR2C	4.71981601	1.0237E-14	3.4124E-15	4.3401E-14
GO:0044459~plasma membrane part	134	16.2424242	3.954E-13	SLC22A18, OCLN, AIF1, SLC6A4, FGFRL1, CSPG4, L1CAM, AQP1, CD209B, B2M, DYSF, PRRT1, SLC2A1, ROBO4, INADL, GBP5, H2-DMB1, TNFRSF14, CD40, SSPN, THY1, JUP, TNS3, RND1, TNS1, PGM5, PTRF, CCR5, DLL4, GM10499, GBP2, HS3ST3B1, CAV2, CAV1, MILL2, ADORA2A, KLRK1, EPHB4, CD74, RIMS3, SLCO2A1, GM8810, IL12RB1, CD69, TEK, H2-T10, SLC28A2, CD8B1, H2-Q5, ACY3, MAOA, H2-Q1, H2-Q6, SLCO2B1, H2-Q7, FCGR1, ABCG1, CAMK2N1, SHANK3, H2-EB2, PKP3, NOTCH4, CD274, PRICKLE2, H2-EB1, H2-T22, H2-T23, SYTL3, H2-T24, CACNA1D, IER3, STEAP4, LIMA1, ART2B, CD8A, SLC15A2, LEPR, CLDN5, BCAM, SYNGR1, CD1D1, GJA4, TENC1, CD1D2, EPCAM, PCDH1, RHOC, SCN7A, NRG1, ATP6V0D2, GPC1, GPIHBP1, LAG3, H2-K1, GPR97, MMP15, MMP14, SLC9A3R2, H2-BL, LCK, CLDN1, KCTD12B, H2-AA, SGCE, RSAD2, KIT, CD24A, CDH5, LY6A, SORBS3, MARVELD2, TGM1, TNFRSF18, CD2, CD4, IL2RG, SLCO3A1, AMICA1, CD5, KCNE3, LY6C1, MS4A4B, ICA1, IL2RB, IL2RA, H2-M3, SLC12A2, OLR1, SELL, H2-AB1, LY6C2, VWF, KCNN4, C920025E04RIK, FCGR2B, HBEGF, JAK2, PERP, TJP2, HTR2C	1.85542091	1.2572E-10	3.143E-11	5.33E-10

GO:0045087~innate immune response	26	3.15151515	1.2456E-12	IFIH1, C3, TLR1, C1S, SP110, CD1D1, C1QC, TLR9, MIF, CD1D2, C1RA, SLC11A1, TMEM173, NOD2, MX1, MX2, DHX58, TLR12, IRGM1, IL18RAP, CFB, C4B, IL27, SAMHD1, FCGR1, C1QB, C1RL	5.8232046	3.2425E-09	4.6321E-10	2.2076E-09
GO:0048002~antigen processing and presentation of peptide antigen	16	1.93939394	2.4654E-12	H2-K1, H2-M3, H2-Q1, H2-DMB1, H2-AB1, H2-Q6, H2-Q7, FCGR1, CD74, B2M, SLC11A1, FCGR2B, GM10499, TAP2, H2-EB1, H2-AA, H2-T23, TAPBPL	10.9553036	6.4173E-09	8.0217E-10	4.3691E-09
GO:0048584~positive regulation of response to stimulus	33	4	6.6194E-12	IGHG1, ADORA2B, C3, KLRK1, C1S, CD1D1, CD24A, C1QC, PNP, SRC, ADA, CD1D2, B2M, C1RA, SLC11A1, SH2D1A, NOD2, TAP2, TGM2, LAG3, H2-K1, H2-M3, C4B, CFB, H2-Q1, H2-Q6, H2-Q7, FCGR1, THY1, C1QB, LAX1, LCK, C1RL, H2-AA, FABP4, JAK2	4.25180634	1.723E-08	1.9145E-09	1.1731E-08
GO:0005615~extracellular space	60	7.27272727	8.6042E-12	IGHG1, GDF3, MSR1, IL18, LEPR, EDN1, TNFSF14, CXCL11, CXCL10, RSPO1, TGFBI, IFNG, PRRT1, SEMA3C, IL15RA, GPIHBP1, GPC1, LTA, EGFL7, C4B, IL27, SAA3, IL22, THBD, IL12A, PLA2G7, IL1F9, CSF2, CXCL5, C3, CSF1, CXCL9, CCL8, PF4, CX3CL1, CCL5, TRF, MIF, CCL6, CCL24, PRSS8, ARG1, IL17A, IL17F, ANGPTL4, SPP1, LPL, BMP2, CCL17, CCL12, ORM1, AFP, AY761185, CXCL13, CXCL16, NOTCH4, C1RL, HBEGF, GDF15, BMP6	2.65493678	2.7361E-09	5.4723E-10	1.16E-08
GO:0050778~positive regulation of immune response	28	3.39393939	9.6343E-12	IGHG1, C3, KLRK1, C1S, CD1D1, CD24A, C1QC, PNP, ADA, CD1D2, B2M, C1RA, SLC11A1, SH2D1A, NOD2, TAP2, LAG3, H2-K1, H2-M3, CFB, C4B, H2-Q1, H2-Q6, H2-Q7, FCGR1, THY1, C1QB, LAX1, LCK, C1RL, H2-AA	4.93391431	2.5078E-08	2.5078E-09	1.7074E-08
GO:0005125~cytokine activity	32	3.87878788	1.3978E-11	GDF3, CSF2, IL1F9, CXCL5, IL18, CSF1, CXCL9, TNFSF14, CCL8, PF4, CX3CL1, CCL5, CXCL11, MIF, CCL6, CXCL10, CCL24, IL17A, IFNG, IL17F, LTA, SPP1, BMP2, IL27, IL22, CCL17, CCL12, CXCL13, CXCL16, IL12A, GDF15, BMP6	4.25641642	1.019E-08	1.019E-08	2.1193E-08
GO:0019884~antigen processing and presentation of exogenous antigen	14	1.6969697	2.0902E-11	H2-K1, H2-M3, H2-DMB1, H2-AB1, CD1D1, FCGR1, CD74, CD1D2, B2M, FCGR2B, PSME1, PSME2, TAP2, H2-EB1, H2-AA	11.9823633	5.4409E-08	4.9463E-09	3.7043E-08
GO:0044421~extracellular region part	77	9.33333333	2.4346E-11	IGHG1, GDF3, MSR1, PODNL1, LEPR, LTBP4, IL18, EDN1, TNFSF14, CXCL11, MMP25, CXCL10, RSPO1, HPSE, TGFBI, IFNG, PRRT1, SEMA3C, IL15RA, GPC1, GPIHBP1, LTA, SPON1, MATN2, EGFL7, C4B, IL27, SAA3, MMP15, MMP14, IL22, THBD, IL12A, PLA2G7, MFAP2, IL1F9, CSF2, CXCL5, C3, CSF1, CXCL9, CCL8, PF4, CX3CL1, CCL5, TRF, CCL6, TIMP1, MIF, PRSS8, CCL24, ARG1, LGALS3BP, IL17A, IL17F, TGM2, SPP1, ANGPTL4, LPL, BMP2, COL4A1, NTN4, NTN1, CCL17, VWF, ORM1, AFP, CCL12, AY761185, CXCL13, CXCL16, KAZALD1, NOTCH4, C1RL, HBEGF, GDF15, BMP6	2.24943578	7.7421E-09	1.2904E-09	3.2823E-08
GO:0042611~MHC protein complex	18	2.18181818	3.7491E-11	H2-K1, H2-Q5, MILL2, H2-M3, H2-Q1, H2-DMB1, H2-AB1, CD1D1, H2-Q6, H2-Q7, CD1D2, B2M, GM8810, H2-EB2, C920025E04RIK, GM10499, H2-BL, H2-EB1, H2-T22, H2-AA, H2-T23, H2-T10, H2-T24	7.82695785	1.1922E-08	1.7032E-09	5.0545E-08
GO:0002703~regulation of leukocyte mediated immunity	20	2.42424242	3.8452E-11	IGHG1, H2-K1, ADORA2B, H2-M3, C3, H2-Q1, KLRK1, CD40, CD1D1, H2-Q6, PNP, CD24A, H2-Q7, FCGR1, CD1D2, B2M, GM8810, NOD2, SH2D1A, FCGR2B, H2-BL, TAP2, IFNG, LAG3	6.84706475	1.0009E-07	8.3409E-09	6.8145E-08
GO:0042127~regulation of cell proliferation	58	7.03030303	6.7729E-11	PTGS2, MARCKSL1, IL18, FGFRL1, PPARG, EDN1, SLFN3, PNP, SLFN1, CXCL10, SPRY1, IFNG, PRRT1, NOS2, CALCRL, LTA, IL27, EFNB1, RASIP1, TNFRSF14, CD40, PDCD1LG2, VASH1, HES1, CD38, TNS3, IL12A, EIF2AK2, CSF2, CAV2, CAV1, ADORA2A, CSF1, IFITM3, PML, KIT, CD24A, CDH5, ADA, MIF, CD9, ALDH1A2, BCL11B, TEK, TGM2, RUNX3, BMP2, IL2RA, TBX3, CRIP2, NTN1, SOD2, FCGR2B, NOTCH4, CD274, FABP4, HBEGF, JAK2	2.58355789	1.763E-07	1.3561E-08	1.2003E-07
GO:0050863~regulation of T cell activation	24	2.90909091	7.5802E-11	ADORA2A, IL18, TNFSF14, CD1D1, CD24A, PNP, CD74, ADA, CD1D2, IFNG, IL2RG, CD4, CD5, LAG3, IL2RA, IL27, EFNB1, TNFRSF14, PDCD1LG2, THY1, TIGIT, CD274, LCK, IL12A, H2-AA	5.32549481	1.9731E-07	1.4094E-08	1.3434E-07

GO:0002706~regulation of lymphocyte mediated immunity	19	2.3030303	8.7524E-11	IGHG1, H2-K1, H2-M3, C3, H2-Q1, KLRK1, CD40, CD1D1, H2-Q6, PNP, H2-Q7, CD24A, FCGR1, CD1D2, B2M, GM8810, NOD2, SH2D1A, FCGR2B, H2-BL, TAP2, IFNG, LAG3	7.00507394	2.2783E-07	1.5188E-08	1.5511E-07
GO:0002694~regulation of leukocyte activation	28	3.39393939	1.9278E-10	ADORA2B, ADORA2A, IL18, TNFSF14, CD1D1, CD24A, PNP, ADA, CD74, CD1D2, IFNG, CD4, IL2RG, CD5, LAG3, IL2RA, IL27, EFNB1, TNFRSF14, CD40, PDCD1LG2, THY1, TIGIT, CD38, FCGR2B, CD274, LCK, IL12A, H2-AA	4.35722302	5.0181E-07	3.1363E-08	3.4165E-07
GO:0051249~regulation of lymphocyte activation	27	3.27272727	2.1464E-10	ADORA2A, IL18, TNFSF14, CD1D1, CD24A, PNP, ADA, CD74, CD1D2, IFNG, CD4, IL2RG, CD5, LAG3, IL2RA, IL27, EFNB1, TNFRSF14, CD40, PDCD1LG2, THY1, TIGIT, CD38, FCGR2B, CD274, LCK, IL12A, H2-AA	4.49338624	5.587E-07	3.2865E-08	3.8038E-07
GO:0002699~positive regulation of immune effector process	16	1.93939394	2.5375E-10	IGHG1, H2-K1, ADORA2B, H2-M3, C3, H2-Q1, KLRK1, CD1D1, H2-Q6, PNP, H2-Q7, CD24A, FCGR1, CD1D2, B2M, NOD2, SH2D1A, TAP2, LAG3	8.33555709	6.6052E-07	3.6696E-08	4.497E-07
GO:0050865~regulation of cell activation	28	3.39393939	2.6106E-10	ADORA2B, ADORA2A, IL18, TNFSF14, CD1D1, CD24A, PNP, ADA, CD74, CD1D2, IFNG, CD4, IL2RG, CD5, LAG3, IL2RA, IL27, EFNB1, TNFRSF14, CD40, PDCD1LG2, THY1, TIGIT, CD38, FCGR2B, CD274, LCK, IL12A, H2-AA	4.30136119	6.7953E-07	3.5765E-08	4.6264E-07
GO:0002705~positive regulation of leukocyte mediated immunity	15	1.81818182	3.4444E-10	IGHG1, H2-K1, H2-M3, C3, H2-Q1, KLRK1, CD1D1, H2-Q6, PNP, H2-Q7, CD24A, FCGR1, CD1D2, B2M, NOD2, SH2D1A, TAP2, LAG3	8.98677249	8.9657E-07	4.4828E-08	6.1041E-07
GO:0002708~positive regulation of lymphocyte mediated immunity	15	1.81818182	3.4444E-10	IGHG1, H2-K1, H2-M3, C3, H2-Q1, KLRK1, CD1D1, H2-Q6, PNP, H2-Q7, CD24A, FCGR1, CD1D2, B2M, NOD2, SH2D1A, TAP2, LAG3	8.98677249	8.9657E-07	4.4828E-08	6.1041E-07
GO:0002821~positive regulation of adaptive immune response	14	1.6969697	4.1129E-10	IGHG1, H2-K1, H2-M3, C3, H2-Q1, CD1D1, H2-Q6, PNP, H2-Q7, CD24A, FCGR1, ADA, CD1D2, B2M, SLC11A1, NOD2, TAP2	9.86782861	1.0706E-06	5.098E-08	7.2889E-07
GO:0002824~positive regulation of adaptive immune response based on somatic recombination of immune receptors built from immunoglobulin superfamily domains	14	1.6969697	4.1129E-10	IGHG1, H2-K1, H2-M3, C3, H2-Q1, CD1D1, H2-Q6, PNP, H2-Q7, CD24A, FCGR1, ADA, CD1D2, B2M, SLC11A1, NOD2, TAP2	9.86782861	1.0706E-06	5.098E-08	7.2889E-07
GO:0002822~regulation of adaptive immune response based on somatic recombination of immune receptors built from immunoglobulin superfamily domains	17	2.06060606	5.8961E-10	IGHG1, H2-K1, H2-M3, C3, H2-Q1, CD40, CD1D1, H2-Q6, PNP, H2-Q7, CD24A, FCGR1, ADA, CD1D2, B2M, SLC11A1, NOD2, FCGR2B, TAP2, IFNG	7.2750063	1.5348E-06	6.9762E-08	1.0449E-06
GO:0002819~regulation of adaptive immune response	17	2.06060606	5.8961E-10	IGHG1, H2-K1, H2-M3, C3, H2-Q1, CD40, CD1D1, H2-Q6, PNP, H2-Q7, CD24A, FCGR1, ADA, CD1D2, B2M, SLC11A1, NOD2, FCGR2B, TAP2, IFNG	7.2750063	1.5348E-06	6.9762E-08	1.0449E-06
GO:0050870~positive regulation of T cell activation	18	2.18181818	1.0143E-09	IL2RA, EFNB1, IL18, TNFSF14, CD1D1, PNP, CD24A, ADA, CD74, PDCD1LG2, CD1D2, THY1, IFNG, LCK, IL12A, H2-AA, CD4, IL2RG, CD5	6.53583454	2.6402E-06	1.1479E-07	1.7975E-06
GO:0002460~adaptive immune response based on somatic recombination of immune receptors built from immunoglobulin superfamily domains	20	2.42424242	1.1597E-09	IGHG1, CD8A, C3, C4B, CFB, IL18, C1S, C1QC, FCGR1, CD74, C1QB, SLC11A1, C1RA, NOD2, IL18BP, FCGR2B, IRF7, C1RL, BCL3, H2-AA	5.70588729	3.0188E-06	1.2578E-07	2.0553E-06
GO:0002250~adaptive immune response	20	2.42424242	1.1597E-09	IGHG1, CD8A, C3, C4B, CFB, IL18, C1S, C1QC, FCGR1, CD74, C1QB, SLC11A1, C1RA, NOD2, IL18BP, FCGR2B, IRF7, C1RL, BCL3, H2-AA	5.70588729	3.0188E-06	1.2578E-07	2.0553E-06
GO:0008009~chemokine activity	14	1.6969697	2.0752E-09	CXCL5, CXCL9, CCL8, PF4, CX3CL1, CXCL11, CCL5, CCL17, CXCL10, CCL6, CCL24, CCL12, CXCL13, CXCL16	8.82086297	1.5128E-06	7.564E-07	3.1465E-06
GO:0002697~regulation of immune effector process	20	2.42424242	2.6771E-09	IGHG1, H2-K1, ADORA2B, H2-M3, C3, H2-Q1, KLRK1, CD40, CD1D1, H2-Q6, PNP, CD24A, H2-Q7, FCGR1, CD1D2, B2M, GM8810, NOD2, SH2D1A, FCGR2B, H2-BL, TAP2, IFNG, LAG3	5.44652878	6.9686E-06	2.7874E-07	4.7444E-06
GO:0042379~chemokine receptor binding	14	1.6969697	2.9954E-09	CXCL5, CXCL9, CCL8, PF4, CX3CL1, CXCL11, CCL5, CCL17, CXCL10, CCL6, CCL24, CCL12, CXCL13, CXCL16	8.59468699	2.1836E-06	7.2788E-07	4.5418E-06
GO:0002696~positive regulation of leukocyte activation	21	2.54545455	3.5062E-09	IL2RA, ADORA2B, EFNB1, IL18, TNFSF14, CD40, CD1D1, PNP, CD24A, ADA, CD74, PDCD1LG2, CD1D2, THY1, CD38, IFNG, LCK, IL12A, H2-AA, CD4, IL2RG, CD5	5.08342686	9.1266E-06	3.5103E-07	6.2137E-06

GO:0051251~positive regulation of lymphocyte activation	20	2.42424242	8.5707E-09	IL2RA, EFNB1, IL18, TNFSF14, CD40, CD1D1, PNP, CD24A, ADA, CD74, PDCD1LG2, CD1D2, THY1, CD38, IFNG, LCK, IL12A, H2-AA, CD4, IL2RG, CD5	5.09887801	2.2309E-05	7.9677E-07	1.5189E-05
GO:0002478~antigen processing and presentation of exogenous peptide antigen	11	1.33333333	1.0184E-08	H2-K1, H2-M3, FCGR2B, TAP2, H2-EB1, H2-AA, H2-DMB1, H2-AB1, FCGR1, CD74, B2M	11.461391	2.651E-05	9.1414E-07	1.8049E-05
GO:0002449~lymphocyte mediated immunity	18	2.18181818	1.0453E-08	IGHG1, CD8A, C3, C4B, CFB, C1S, C1QC, FCGR1, CD74, C1QB, SLC11A1, C1RA, NOD2, FCGR2B, IRF7, C1RL, BCL3, H2-AA	5.67585631	2.7209E-05	9.0697E-07	1.8525E-05
GO:0016064~immunoglobulin mediated immune response	16	1.93939394	3.1395E-08	IGHG1, C3, C4B, CFB, C1S, C1QC, FCGR1, CD74, C1QB, C1RA, NOD2, FCGR2B, IRF7, C1RL, BCL3, H2-AA	6.08627978	8.1719E-05	2.6362E-06	5.5639E-05
GO:0019724~B cell mediated immunity	16	1.93939394	4.9347E-08	IGHG1, C3, C4B, CFB, C1S, C1QC, FCGR1, CD74, C1QB, C1RA, NOD2, FCGR2B, IRF7, C1RL, BCL3, H2-AA	5.89900963	0.00012844	4.0141E-06	8.7453E-05
GO:0002252~immune effector process	22	2.66666667	5.1112E-08	IGHG1, CD8A, C3, C4B, CFB, H2-Q1, SAMHD1, RSAD2, C1S, H2-Q6, FCGR1, C1QC, H2-Q7, CD74, C1QB, SLC11A1, C1RA, NOD2, FCGR2B, LAX1, IRF7, C1RL, H2-AA, BCL3	4.18431735	0.00013304	4.0316E-06	9.058E-05
GO:0042330~taxis	20	2.42424242	1.0604E-07	CXCL5, FPR1, CCL8, CXCR1, PF4, CX3CL1, KIT, CXCL11, CCL5, CCL17, CCL6, CXCL10, CCL24, CCL12, CXCL13, CXCL16, SEMA3F, IFNG, ECSCR, PTAFR	4.39719755	0.00027598	8.1181E-06	0.00018792
GO:0006935~chemotaxis	20	2.42424242	1.0604E-07	CXCL5, FPR1, CCL8, CXCR1, PF4, CX3CL1, KIT, CXCL11, CCL5, CCL17, CCL6, CXCL10, CCL24, CCL12, CXCL13, CXCL16, SEMA3F, IFNG, ECSCR, PTAFR	4.39719755	0.00027598	8.1181E-06	0.00018792
GO:0012502~induction of programmed cell death	25	3.03030303	1.0861E-07	IGHG1, ADORA2A, IL18, CIDEB, PML, NR4A1, GZMB, SP110, FCGR1, CD24A, CIDEC, PLEKHF1, CASP4, LCK, CASP12, PYCARD, RIPK3, TGM2, BCL3, BIK, PERP, CD5, LTA, RUNX3, IFI204	3.58753393	0.00028268	8.0776E-06	0.00019248
GO:0006917~induction of apoptosis	25	3.03030303	1.0861E-07	IGHG1, ADORA2A, IL18, CIDEB, PML, NR4A1, GZMB, SP110, FCGR1, CD24A, CIDEC, PLEKHF1, CASP4, LCK, CASP12, PYCARD, RIPK3, TGM2, BCL3, BIK, PERP, CD5, LTA, RUNX3, IFI204	3.58753393	0.00028268	8.0776E-06	0.00019248
GO:0002443~leukocyte mediated immunity	18	2.18181818	1.2521E-07	IGHG1, CD8A, C3, C4B, CFB, C1S, C1QC, FCGR1, CD74, C1QB, SLC11A1, C1RA, NOD2, FCGR2B, IRF7, C1RL, BCL3, H2-AA	4.84679864	0.00032587	9.0533E-06	0.0002219
GO:0042612~MHC class I protein complex	13	1.57575758	1.5969E-07	H2-K1, MILL2, H2-Q5, H2-M3, H2-Q1, CD1D1, H2-Q6, H2-Q7, CD1D2, B2M, GM8810, C920025E04RIK, H2-BL, GM10499, H2-T22, H2-T10, H2-T23, H2-T24	6.99870834	5.0781E-05	6.3478E-06	0.00021529
GO:0001912~positive regulation of leukocyte mediated cytotoxicity	10	1.21212121	1.7157E-07	H2-K1, SH2D1A, H2-M3, TAP2, H2-Q1, KLRK1, CD1D1, H2-Q6, H2-Q7, PNP, LAG3, CD1D2, B2M	10.4194464	0.00044651	1.207E-05	0.00030406
GO:0031343~positive regulation of cell killing	10	1.21212121	1.7157E-07	H2-K1, SH2D1A, H2-M3, TAP2, H2-Q1, KLRK1, CD1D1, H2-Q6, H2-Q7, PNP, LAG3, CD1D2, B2M	10.4194464	0.00044651	1.207E-05	0.00030406
GO:0001944~vasculature development	31	3.75757576	1.808E-07	CAV1, PGF, LEPR, IL18, EDN1, CSPG4, ANPEP, CX3CL1, GJA4, CDH5, ALDH1A2, WARS, HEY1, ROBO4, TGM2, SEMA3C, SOX18, NOS2, TBX3, EGFL7, EPAS1, SOCS3, EFNB2, MMP14, VASH1, THY1, HIF1A, DLL4, NOTCH4, ECSCR, HBEGF	2.9716261	0.00047052	1.2385E-05	0.00032042

GO:0005576~extracellular region	118	14.3030303	1.8233E-07	GDF3, PGF, LTBP4, IL18, EDN1, TNFSF14, RETNLG, CXCL11, C1QC, CXCL10, B2M, ISG15, PGLYRP2, IFNG, TGFBI, PRRT1, IL15RA, SPON1, MATN2, F10, C4B, IL27, CD40, IL22, VASH1, C1QB, THBD, WFDC10, CST7, WFDC1, MFAP2, CCL8, CX3CL1, CCL5, TRF, CCL6, TIMP1, ARG1, LGALS3BP, ACE, FOLR4, IL17A, IL17F, FGL2, PLTP, ANGPTL4, SPP1, LPL, BMP2, CFB, NTN4, TINAGL1, NTN1, SECTM1A, LCN2, AY761185, CXCL13, CXCL16, NOTCH4, BMP6, IGHG1, MSR1, PODNL1, LEPR, SPINK2, HP, INSL6, MMP25, RSPO1, HPSE, SEMA3G, SEMA3F, SEMA3E, SEMA3C, GPC1, GPIHBP1, LTA, EGFL7, SAA3, MMP15, MMP14, MCPT8, SERPINA3N, IL18BP, IL12A, PLA2G7, SLPI, IL1F9, CSF2, CXCL5, C3, CSF1, CXCL9, PF4, MIF, PRSS8, CCL24, C1QTNF6, TNFRSF18, CD2, PYCARD, TGM2, VSTM2A, COL4A1, OLR1, CCL17, VWF, AFP, CCL12, ORM1, FCGR2B, KAZALD1, C1RL, HBEGF, EPOR, GDF15, LIPF, ENHO	1.58816843	5.7978E-05	6.4422E-06	0.00024581
GO:0031341~regulation of cell killing	11	1.33333333	2.0532E-07	H2-K1, H2-M3, H2-Q1, KLRK1, H2-Q6, CD1D1, PNP, H2-Q7, B2M, CD1D2, GM8810, SH2D1A, TAP2, H2-BL, LAG3	8.78706643	0.00053431	1.3704E-05	0.00036387
GO:0001910~regulation of leukocyte mediated cytotoxicity	11	1.33333333	2.0532E-07	H2-K1, H2-M3, H2-Q1, KLRK1, H2-Q6, CD1D1, PNP, H2-Q7, B2M, CD1D2, GM8810, SH2D1A, TAP2, H2-BL, LAG3	8.78706643	0.00053431	1.3704E-05	0.00036387
GO:0002711~positive regulation of T cell mediated immunity	8	0.96969697	2.8919E-07	H2-K1, H2-M3, TAP2, H2-Q1, CD1D1, H2-Q6, H2-Q7, CD24A, PNP, CD1D2, B2M	14.7475241	0.00075249	1.8819E-05	0.00051251
GO:0001568~blood vessel development	30	3.63636364	3.5514E-07	CAV1, PGF, LEPR, IL18, EDN1, CSPG4, ANPEP, CX3CL1, GJA4, CDH5, ALDH1A2, WARS, HEY1, ROBO4, TGM2, SEMA3C, SOX18, NOS2, TBX3, EGFL7, EPAS1, SOCS3, MMP14, VASH1, THY1, HIF1A, DLL4, NOTCH4, ECSCR, HBEGF	2.94648278	0.000924	2.2547E-05	0.00062937
GO:0001916~positive regulation of T cell mediated cytotoxicity	7	0.84848485	3.84E-07	H2-K1, H2-M3, TAP2, H2-Q1, CD1D1, H2-Q6, H2-Q7, PNP, CD1D2, B2M	18.6392318	0.00099905	2.3799E-05	0.00068053
GO:0051240~positive regulation of multicellular organismal process	23	2.78787879	1.1002E-06	CAV1, PTPRM, ADORA2B, H2-M3, ADORA2A, CSF1, IL18, EDN1, H2-Q1, KLRK1, CX3CL1, CD40, H2-Q6, H2-Q7, ADA, TRF, TIGIT, SLC11A1, NOD2, ADRB1, IFNG, PYCARD, BCL3, HTR2C, LTA	3.38152584	0.00285969	6.6598E-05	0.00194974
GO:0001817~regulation of cytokine production	21	2.54545455	1.2219E-06	ADORA2B, H2-M3, IL18, PPARG, H2-Q1, TLR1, KLRK1, CX3CL1, CD40, CD1D1, CD209B, H2-Q6, CD24A, H2-Q7, TLR9, CD1D2, TIGIT, SLC11A1, NOD2, IFNG, PYCARD, IRF1, BCL3, LAG3	3.62057021	0.00317568	7.2287E-05	0.00216552
GO:0006959~humoral immune response	13	1.57575758	1.6775E-06	IGHG1, C1QB, C1RA, SH2D1A, C3, C4B, CFB, CCR2, C1RL, BCL3, C1S, C1QC, LTA	5.76928604	0.00435695	9.7028E-05	0.00297279
GO:0001914~regulation of T cell mediated cytotoxicity	7	0.84848485	1.9657E-06	H2-K1, H2-M3, TAP2, H2-Q1, CD1D1, H2-Q6, H2-Q7, PNP, CD1D2, B2M	15.2502806	0.0051037	0.00011123	0.00348359
GO:0009617~response to bacterium	22	2.66666667	2.2336E-06	IGHG1, H2-K1, H2-M3, TLR1, SP110, STAT1, CD209B, CD24A, FCGR1, B2M, SLC11A1, NOD2, THBD, CCR5, TAP2, PGLYRP2, IFNG, IRF8, BCL3, NOS2, IRG1, PTAFR	3.35811456	0.00579711	0.00012369	0.00395826
GO:0008285~negative regulation of cell proliferation	27	3.27272727	2.2437E-06	CAV2, CAV1, PTGS2, ADORA2A, IFITM3, FGFRL1, PPARG, PML, SLFN3, CD24A, CDH5, SLFN1, CD9, SPRY1, BCL11B, LTA, RUNX3, BMP2, IL2RA, TNFRSF14, VASH1, PDCD1LG2, SOD2, FCGR2B, CD274, JAK2, EIF2AK2	2.88860544	0.00582343	0.00012167	0.00397628
GO:0001525~angiogenesis	20	2.42424242	2.5119E-06	EPAS1, EGFL7, PGF, LEPR, IL18, EDN1, CSPG4, ANPEP, CX3CL1, MMP14, VASH1, THY1, WARS, HIF1A, DLL4, NOTCH4, ECSCR, ROBO4, HBEGF, SOX18	3.60371829	0.00651716	0.00013343	0.00445151
GO:0009615~response to virus	15	1.81818182	2.5531E-06	IFIH1, H2-Q1, SAMHD1, RSAD2, H2-Q6, H2-Q7, TLR9, LCN2, ISG15, IFI27L2A, IRF7, IFNG, OAS1B, OAS1A, EIF2AK2, MX1, MX2	4.72988026	0.00662366	0.00013291	0.00452449
GO:0048514~blood vessel morphogenesis	25	3.03030303	2.5603E-06	CAV1, EGFL7, EPAS1, PGF, IL18, LEPR, EDN1, CSPG4, ANPEP, CX3CL1, MMP14, VASH1, THY1, WARS, HIF1A, HEY1, DLL4, NOTCH4, ECSCR, ROBO4, TGM2, SEMA3C, HBEGF, SOX18, NOS2	3.02584932	0.00664234	0.00013067	0.00453729

GO:0008284~positive regulation of cell proliferation	31	3.75757576	2.7371E-06	CSF2, MARCKSL1, IL18, CSF1, EDN1, KIT, CD24A, PNP, ADA, CXCL10, ALDH1A2, IFNG, PRRT1, TGM2, CALCRL, LTA, IL2RA, TBX3, CRIP2, EFNB1, RASIP1, CD40, NTN1, PDCD1LG2, HES1, CD38, TNS3, NOTCH4, IL12A, HBEGF, FABP4	2.61586805	0.00709925	0.000137	0.00485051
GO:0043067~regulation of programmed cell death	48	5.81818182	3.6844E-06	IGHG1, XRCC5, TRAF1, CSF2, IFIH1, ADORA2A, IL18, PML, CX3CL1, KIT, SP110, PNP, CD24A, CD74, ADA, SRC, TIMP1, NOD2, CASP4, BCL11B, TGM2, PYCARD, BCL3, CD5, LTA, RUNX3, ANGPTL4, SPP1, IL2RB, IL2RA, TBX3, CIDEB, NR4A1, GZMB, PIM2, FCGR1, SOD2, CIDEC, CARD10, PLEKHF1, HIF1A, LCK, CASP12, RIPK3, BIK, ID3, PERP, IFI204	2.05411943	0.00954459	0.00018094	0.00652925
GO:0042325~regulation of phosphorylation	31	3.75757576	4.19E-06	CAV1, ADORA2A, EFNA1, CSF1, EDN1, TLR1, CSPG4, KIT, CD24A, APLP2, CD74, PRKAR2B, SLC11A1, SPRY1, NOD2, IFNG, CD4, NRG1, FAM129A, CEACAM1, PPP1R14A, BMP2, SOCS3, SOCS1, TNFRSF14, IL22, THY1, CARD10, CDKN1C, FABP4, JAK2	2.56174664	0.01084729	0.00020195	0.00742524
GO:0010941~regulation of cell death	48	5.81818182	4.2366E-06	IGHG1, XRCC5, TRAF1, CSF2, IFIH1, ADORA2A, IL18, PML, CX3CL1, KIT, SP110, PNP, CD24A, CD74, ADA, SRC, TIMP1, NOD2, CASP4, BCL11B, TGM2, PYCARD, BCL3, CD5, LTA, RUNX3, ANGPTL4, SPP1, IL2RB, IL2RA, TBX3, CIDEB, NR4A1, GZMB, PIM2, FCGR1, SOD2, CIDEC, CARD10, PLEKHF1, HIF1A, LCK, CASP12, RIPK3, BIK, ID3, PERP, IFI204	2.04317385	0.01096726	0.00020049	0.00750782
GO:0002709~regulation of T cell mediated immunity	8	0.96969697	4.4682E-06	H2-K1, H2-M3, TAP2, H2-Q1, CD1D1, H2-Q6, H2-Q7, CD24A, PNP, CD1D2, B2M	10.6509896	0.0115633	0.00020767	0.00791821
GO:0050670~regulation of lymphocyte proliferation	15	1.81818182	4.8058E-06	IL2RA, IL27, IL18, EFNB1, TNFRSF14, CD40, PNP, CD24A, PDCD1LG2, ADA, CD38, FCGR2B, CD274, IFNG, IL12A	4.49338624	0.01243158	0.00021944	0.00851649
GO:0032944~regulation of mononuclear cell proliferation	15	1.81818182	4.8058E-06	IL2RA, IL27, IL18, EFNB1, TNFRSF14, CD40, PNP, CD24A, PDCD1LG2, ADA, CD38, FCGR2B, CD274, IFNG, IL12A	4.49338624	0.01243158	0.00021944	0.00851649
GO:0043065~positive regulation of apoptosis	28	3.39393939	4.9005E-06	IGHG1, ADORA2A, IL18, PML, SP110, CD24A, SRC, CASP4, PYCARD, TGM2, BCL3, CD5, RUNX3, LTA, IL2RA, CIDEB, NR4A1, GZMB, FCGR1, CIDEC, PLEKHF1, CASP12, LCK, RIPK3, BIK, ID3, PERP, IFI204	2.70569494	0.01267506	0.00021991	0.00868435
GO:0043068~positive regulation of programmed cell death	28	3.39393939	5.7192E-06	IGHG1, ADORA2A, IL18, PML, SP110, CD24A, SRC, CASP4, PYCARD, TGM2, BCL3, CD5, RUNX3, LTA, IL2RA, CIDEB, NR4A1, GZMB, FCGR1, CIDEC, PLEKHF1, CASP12, LCK, RIPK3, BIK, ID3, PERP, IFI204	2.68404938	0.01477694	0.00025229	0.01013516
GO:0042981~regulation of apoptosis	47	5.6969697	5.9112E-06	IGHG1, XRCC5, TRAF1, CSF2, IFIH1, ADORA2A, IL18, PML, CX3CL1, SP110, PNP, CD24A, CD74, ADA, SRC, TIMP1, NOD2, CASP4, BCL11B, TGM2, PYCARD, BCL3, CD5, LTA, RUNX3, ANGPTL4, SPP1, IL2RB, IL2RA, TBX3, CIDEB, NR4A1, GZMB, PIM2, FCGR1, SOD2, CIDEC, CARD10, PLEKHF1, HIF1A, LCK, CASP12, RIPK3, BIK, ID3, PERP, IFI204	2.03678508	0.01526917	0.00025642	0.01047536
GO:0070663~regulation of leukocyte proliferation	15	1.81818182	6.4891E-06	IL2RA, IL27, IL18, EFNB1, TNFRSF14, CD40, PNP, CD24A, PDCD1LG2, ADA, CD38, FCGR2B, CD274, IFNG, IL12A	4.38379146	0.01674933	0.00027687	0.01149938
GO:0010942~positive regulation of cell death	28	3.39393939	6.6166E-06	IGHG1, ADORA2A, IL18, PML, SP110, CD24A, SRC, CASP4, PYCARD, TGM2, BCL3, CD5, RUNX3, LTA, IL2RA, CIDEB, NR4A1, GZMB, FCGR1, CIDEC, PLEKHF1, CASP12, LCK, RIPK3, BIK, ID3, PERP, IFI204	2.6627474	0.01707558	0.00027775	0.0117253
GO:0007626~locomotory behavior	27	3.27272727	7.418E-06	CXCL5, ADORA2A, FPR1, CXCR1, SOBP, CCL8, PF4, KIT, CX3CL1, CXCL11, CCL5, APLP2, CCL6, CXCL10, CCL24, MCOLN3, SEMA3F, IFNG, NRG1, MYO6, CCL17, SOD2, CCL12, CXCL13, CXCL16, ECSCR, PTAFR	2.70731221	0.01912387	0.00030645	0.01314537
GO:0001819~positive regulation of cytokine production	13	1.57575758	7.7918E-06	ADORA2B, H2-M3, IL18, H2-Q1, KLRK1, CD40, CX3CL1, H2-Q6, H2-Q7, TIGIT, SLC11A1, NOD2, IFNG, PYCARD, BCL3	5.02486204	0.02007778	0.00031686	0.01380772
GO:0019220~regulation of phosphate metabolic process	31	3.75757576	8.8249E-06	CAV1, ADORA2A, EFNA1, CSF1, EDN1, TLR1, CSPG4, KIT, CD24A, APLP2, CD74, PRKAR2B, SLC11A1, SPRY1, NOD2, IFNG, CD4, NRG1, FAM129A, CEACAM1, PPP1R14A, BMP2, SOCS3, SOCS1, TNFRSF14, IL22, THY1, CARD10, CDKN1C, FABP4, JAK2	2.46812799	0.02270947	0.00035334	0.01563835

GO:0051174~regulation of phosphorus metabolic process	31	3.75757576	8.8249E-06	CAV1, ADORA2A, EFNA1, CSF1, EDN1, TLR1, CSPG4, KIT, CD24A, APLP2, CD74, PRKAR2B, SLC11A1, SPRY1, NOD2, IFNG, CD4, NRG1, FAM129A, CEACAM1, PPP1R14A, BMP2, SOCS3, SOCS1, TNFRSF14, IL22, THY1, CARD10, CDKN1C, FABP4, JAK2	2.46812799	0.02270947	0.00035334	0.01563835
GO:0002455~humoral immune response mediated by circulating immunoglobulin	10	1.21212121	9.4685E-06	IGHG1, C1QB, C1RA, C3, C4B, CFB, C1RL, BCL3, C1S, C1QC	6.84706475	0.02434525	0.00037336	0.01677869
GO:0001775~cell activation	27	3.27272727	1.2495E-05	CSF2, CD8A, ADORA2A, TLR1, KLRK1, SOX4, PF4, CD1D1, CD24A, ADA, CD74, HSH2D, CD1D2, TIMP1, SLC11A1, BCL11B, BCL3, CD4, LTA, SATB1, H2-M3, SLA2, CD40, VWF, FCGR2B, LAX1, LCK, IRF1	2.63027487	0.03200116	0.00048532	0.02214116
GO:0070482~response to oxygen levels	13	1.57575758	1.2963E-05	CAV1, EPAS1, IL18, EDN1, PML, MMP14, CD24A, TRF, SOD2, ACE, HIF1A, UCP3, NOS2	4.79294533	0.03318032	0.0004961	0.02297083
GO:0002683~negative regulation of immune system process	14	1.6969697	1.3546E-05	IL2RA, ADORA2A, TNFRSF14, CD24A, ADA, CD74, PDCD1LG2, THY1, TIGIT, GM8810, NOD2, FCGR2B, H2-BL, CD274, LAG3	4.41455491	0.03464661	0.0005109	0.02400393
GO:0010033~response to organic substance	43	5.21212121	1.5147E-05	CAV1, ART2B, ADORA2A, LEPR, PPARG, TLR1, SERPINA3F, CCL5, CD24A, TRF, SRC, ACSBG1, B2M, SLC11A1, NOD2, TAP2, IFNG, CD4, DDAH2, IRG1, EGR2, CYP2D22, H2-M3, GATM, PFKL, CYP11A1, CFB, SELL, MMP14, STAT1, LPIN1, VASH1, SERPINA3N, THBD, SERPINA3H, SERPINA3G, SERPINA3I, CASP12, FABP4, JAK2, ID3, EIF2AK2, HTR2C, PTAFR	2.04056088	0.038661	0.0005631	0.02684032
GO:0019955~cytokine binding	15	1.81818182	1.5205E-05	IL2RB, IL2RA, IL18RAP, LEPR, IL21R, CXCR1, CXCR3, CD74, IL12RB1, IL18BP, CCR5, CCR2, IL12A, EPOR, IL2RG	4.08108108	0.01102366	0.00276738	0.02305288
GO:0031349~positive regulation of defense response	12	1.45454545	1.9169E-05	IGHG1, NOD2, SH2D1A, ADORA2B, H2-M3, C3, KLRK1, TGM2, FABP4, CD24A, FCGR1, LAG3	5.04520561	0.04867186	0.00070252	0.03396513
GO:0050727~regulation of inflammatory response	12	1.45454545	1.9169E-05	IGHG1, IL2RA, ADORA2B, FCGR2B, C3, ADORA2A, AOAH, TGM2, FABP4, CD24A, FCGR1, ADA	5.04520561	0.04867186	0.00070252	0.03396513
GO:0006958~complement activation, classical pathway	9	1.09090909	2.2392E-05	IGHG1, C1QB, C1RA, C3, C4B, CFB, C1RL, C1S, C1QC	7.18941799	0.05662201	0.00080923	0.03967656
GO:0002237~response to molecule of bacterial origin	11	1.33333333	2.7479E-05	SLC11A1, NOD2, THBD, H2-M3, TAP2, TLR1, STAT1, CD24A, IRG1, PTAFR, B2M	5.37983659	0.0690306	0.00097937	0.04868722
GO:0002526~acute inflammatory response	14	1.6969697	2.7516E-05	IGHG1, CFB, C4B, C3, SAA3, C1S, C1QC, FCGR1, TRF, ORM1, C1RA, C1QB, SERPINA3N, C1RL	4.14205152	0.06912056	0.00096744	0.04875298
