# Supplementary material for: IFN-γ immune priming of macrophages in vivo induces prolonged STAT1 binding and protection against Cryptococcus neoformans
Source: PLoS Pathog. 2018 Oct 10;14(10):e1007358. doi: 10.1371/journal.ppat.1007358 (PMC6197699; doi:10.1371/journal.ppat.1007358)
Supplement: S3 Table — Antibodies used for flow cytometric analysis and western blot analysis. (PDF) [file ppat.1007358.s005.pdf]

Supplementary Table 3: Antibodies

Flow Cytometry

Marker	Clone	Source	Catalog #
1A8 (Ly-6G)-APC	1A8	BD Biosciences	560599
Gr1 (Ly6G/Ly6c)-PE	RB6-8C5	BD Biosciences	553128
CD335 (NKp46)-eFluor 660	29A1.4	eBiosciences	50-3351-80
CD3e-PE-Cy7	145-2C11	eBiosciences	25-0031-81
CD3-PE-Cy7	145-2C11	BD Biosciences	T cell Mix, 558431
CD4-PE	RM4-5	BD Biosciences	
CD8a-APC	53-6.7	BD Biosciences	
F4/80-PE	BM8	eBiosciences	12-4801-80
F4/80-Biotin	BM8	eBiosciences	13-4801-82
CD11b-PE	M1/70	Biolegend	101224
CD64-PE-Cy7	X54-5/7.1	Biolegend	139317
TNF-α-PE	MP6-XT22	eBiosciences	12-7321-81
IFN-γ-APC	XMG1.2	eBiosciences	17-7311-81
IL-2-PE	JES6-5H4	BD Biosciences	561061
Labeling Check Reagent-APC	n/a	Miltenyi Biotec	130-095-237

Western Blot

Marker	Clone	Source	Catalog #
STAT1	polyclonal	Cell Signaling	9172
STAT1-phosphorylated Tyr701	58D6	Cell Signaling	9167
Torin	7C10	Cell Signaling	2983
Torin-phosphorylated Ser2448	D9C2	Cell Signaling	5536
β-actin	13E5	Cell Signaling	4970
